# Unveiling the Paradoxical Tumor-Suppressive Role of CCL2/CCR2 in Bladder Cancer: A Novel Immunotherapeutic Strategy

**DOI:** 10.3390/cancers18142267

**Published:** 2026-07-15

**Authors:** Neelam Mukherjee, Niannian Ji, Zaineb Hassouneh, Jaime Furman, Olivia Fisher, Jonathan Gelfond, Onika D. V. Noel, Gisele Morales, Xi Tan, Chun-Liang Chen, Solomon L. Woldu, Yair Lotan, Robert S. Svatek

**Affiliations:** 1 Department of Urology, University of Texas Health San Antonio, San Antonio, TX 78229, USA; jin3@uthscsa.edu (N.J.); hassouneh@uthscsa.edu (Z.H.); noelo@uthscsa.edu (O.D.V.N.); moralesg8@uthscsa.edu (G.M.); 2 Department of Microbiology, Immunology & Molecular Genetics, University of Texas Health San Antonio, San Antonio, TX 78229, USA; 3 Department of Pathology, University of Texas Health San Antonio, San Antonio, TX 78229, USA; jfurman@pathreflab.com (J.F.); fishero1@uthscsa.edu (O.F.); 4 Department of Population Health Sciences, University of Texas Health San Antonio, San Antonio, TX 78229, USA; gelfondjal@uthscsa.edu; 5 Department of Molecular Medicine, University of Texas Health San Antonio, San Antonio, TX 78229, USA; tanx3@uthscsa.edu (X.T.); chenc4@uthscsa.edu (C.-L.C.); 6 Department of Urology, University of Texas Southwestern Medical Center, Dallas, TX 75235, USA; solomon.woldu@utsouthwestern.edu (S.L.W.); yair.lotan@utsouthwestern.edu (Y.L.)

**Keywords:** BCa, CCL2, CCR2, T cells, chemokine

## Abstract

**Background**: Bladder cancer (BCa) is characterized by frequent recurrence and limited durable responses to immunotherapy, in part due to poor T-cell infiltration into tumors. While the chemokine CCL2 and its receptor CCR2 have traditionally been associated with recruitment of immunosuppressive myeloid cells and tumor promotion, we reveal an unexpected anti-tumor role for this pathway in BCa. **Methods**: Using orthotopic and carcinogen-induced murine BCa models, we demonstrate that genetic deletion or antibody blockade of CCL2 or CCR2 accelerates tumor progression, reduces intratumoral CD4^+^ and CD8^+^ T-cell infiltration, and shortens survival. **Results**: Mechanistic studies show that CCL2 promotes recruitment of CCR2^+^ effector T cells with enhanced activation and cytotoxicity. Mixed bone marrow chimeras, T-cell-specific CCR2 knockouts, and adoptive transfers confirm that CCR2 signaling within T cells is essential for their trafficking and anti-tumor function. In human BCa, CCL2 expression is reduced in tumors compared to adjacent urothelium, correlating with diminished T-cell infiltration. Importantly, high tumor CCL2 levels are associated with improved recurrence-free survival in patients with BCa. To therapeutically leverage this pathway, we developed a novel intravesical recombinant CCL2 (rCCL2) approach. rCCL2 delivery significantly increased CCR2^+^ T-cell infiltration, reduced tumor burden, and extended survival in both syngeneic MB49 and double-humanized patient-derived xenograft (PDX) BCa models. **Conclusions**: These findings redefine the CCL2-CCR2 axis as a T-cell-mediated tumor-suppressive pathway in BCa and support rCCL2-based therapy as a strategy to enhance immune infiltration and improve outcomes in treatment-resistant BCa.

## Simple Summary

CCL2–CCR2 signaling has long been associated with tumor-promoting myeloid cell recruitment and poor outcomes across various cancers. However, our findings redefine this paradigm by demonstrating a tissue-specific, anti-tumor role for CCL2 in BCa. Using multiple murine BCa models, we show that loss of CCL2 or CCR2 enhances tumor progression, while intravesical administration of recombinant CCL2 reduces tumor growth and improves survival by promoting CCR2^+^ T-cell recruitment into the bladder tumor microenvironment. Supporting these findings, human BCa tissues display reduced CCL2 expression and lower T-cell infiltration compared to adjacent normal tissues, and higher intratumoral CCL2 levels correlate with improved recurrence-free survival. These findings highlight a context-dependent, immunostimulatory function of CCL2 in BCa and establish the therapeutic potential of harnessing this axis to enhance anti-tumor T-cell immunity.

## 1. Introduction

Bladder cancer (BCa) remains a significant public health challenge [1] due to its high recurrence rates, substantial management costs [2], and resistance to standard treatments [3,4]. The recruitment and activation of immune cells within the bladder tumor microenvironment are key drivers of tumor progression [5] and response to therapy [6], highlighting the need for novel approaches targeting these immune interactions. The homing of immune cells to tumors depends on the concerted action of adhesion molecules and chemokines [7]. Chemokines are small proteins that activate downstream signaling after binding to a subfamily of G-protein-coupled receptors [8]. Chemokines contribute to cancer development and progression by recruiting tumor-promoting or tumor-suppressive immune cells into the tumor environment [9]. This coordinated pattern of chemokine expression regulates the recruitment and activation of immune cells, thereby regulating tumor progression. Consequently, chemokines remain of significant interest as potential drug targets for clinical interventions in cancer.

CCL2 (C-C Motif Chemokine Ligand 2) is a 13 kDa inducible chemokine also known as monocyte chemoattractant protein 1 (MCP-1) due to its chemotactic and stimulatory effects on monocytes [10,11]. Following its original discovery, it was later found to be identical to the tumor cell-derived chemotactic factor, which recruits tumor-associated macrophages (TAMs) into tumors [12]. CCL2 binds primarily to CCR2 (C-C chemokine receptor type 2), resulting in the release of intracellular calcium and activation of downstream signal transduction [13] that drives chemotaxis. CCL2 is commonly known for its chemotactic induction of TAMs and myeloid-derived suppressor cells (MDSCs), which are involved in immune evasion [14,15,16,17,18]. Consequently, increased CCL2 levels are linked to poor prognosis in several cancers [16,19,20,21,22], and CCL2-targeted neutralizing antibodies like the monoclonal antibody carlumab (CNTO 888) [23,24] are being tested in cancer trials [25]. However, CCL2 also recruits anti-tumor immune cells [26,27] and, in some environments, blocking CCL2/CCR2 signaling could have detrimental effects [27,28,29].

The role of CCL2/CCR2 signaling in BCa remains poorly defined. Although correlative studies have linked CCL2 overexpression with tumor progression, suggesting a pro-tumoral role for this axis, other studies indicate that the association between CCL2 expression and mortality is cell context-dependent [30,31]. The mechanistic pro-tumoral function supporting or disproving these findings, however, has not been investigated. To study the CCL2/CCR2 axis in BCa, transgenic and humanized murine models were employed alongside examination of the predictive significance of tumor CCL2 in human clinical tissues. A novel T-cell-dependent, microenvironment-specific, anti-tumor function of CCL2/CCR2 in BCa was discovered. We identify tumor CCL2 expression as a possible prognostic marker in BCa and demonstrate the efficacy of recombinant CCL2, in combination with chemotherapy, in BCa in mouse models.

## 2. Materials and Methods

### 2.1. Human Subjects and Eligibility Criteria

Human subjects were not studied directly; however, studies were performed using the bladder cancer patient tissue inventory where patients were recruited through the UT Health San Antonio Institutional Review Board (IRB)-approved observational cohort study, which collected clinical data and bladder tissue for analysis (IRB # HSC2012-159H). All biospecimens are completely anonymized, well-annotated and linked to pathology and clinical databases.

For the CCL2 immunohistochemical (IHC) staining cohort, patients were retrospectively identified from prospectively collected databases at two tertiary referral centers in the United States (University of Texas Southwestern, UTSW) and Israel (Rabin Medical Center, RMC). Exclusion criteria included previously untreated or concurrently diagnosed upper tract urothelial carcinoma (UTUC), receipt of intravesical bacillus Calmette–Guérin (BCG) treatment in the last three years, and lack of BCG induction. The primary outcome was BCG unresponsiveness, defined by the presence of high-grade disease at 6 months following adequate BCG (one induction and maintenance cycle or two induction cycles) for pT1 or 12 months for CIS or the presence of pT1 at 3 months following induction. BCG failure was defined as any high-grade recurrence following BCG induction. We also evaluated persistent or recurrent high-grade disease following BCG induction that did not necessarily meet the strict definition of BCG unresponsive disease.

All patients underwent initial transurethral resection (TUR) for pathologic diagnosis of urothelial carcinoma and subsequently underwent BCG induction. The use of immediate post-operative intravesical chemotherapy and repeat TUR prior to BCG was determined by the treating clinician. Patients were then placed on a surveillance protocol determined by the treating clinician. TUR was performed based on surveillance cystoscopy findings, which guided subsequent recommendations for continued surveillance or other management.

### 2.2. Mice

Male and female C57BL/6 wildtype (WT; CD45.2) mice (Stock No: 000664) and female CD45.1/C57BL/6 mice at 6–8 weeks of age were obtained from the Jackson Laboratory for experiments. CCL2 knockout (CCL2^−/−^) and CCR2^−/−^ breeders were obtained from the Jackson Laboratory and bred in-house to produce offspring for experiments. TCRα KO, Rag1 KO, and NSG mice were obtained from the Jackson Laboratory and bred in-house. Age-matched male mice were used for N-Butyl-N-(4-hydroxybutyl)-nitrosamine (BBN)-exposure studies, whereas age-matched female mice were used for all subcutaneous (subQ) or orthotopic bladder tumor studies. All mice were maintained under conventional housing conditions except for immunocompromised TCRα KO, Rag1 KO, and NSG mice that were housed in specific pathogen-free conditions. All animal studies were performed using procedures approved by the UT Health San Antonio Institutional Animal Care and Use Committee (Protocol #20120040AR/20150058AR). No criteria were set for including and excluding animals. There were no exclusions of experimental animals, units or data points.

### 2.3. Cell Lines and Culture Conditions

MB49 cells were cultured in complete DMEM (Corning, Manassas, VA, USA; cDMEM-10) media supplemented with 10% fetal bovine serum (FBS; HyClone/Thermo Fisher Scientific, Logan, UT, USA), 2 mM L-glutamine (Corning), and penicillin at 100 IU/mL plus streptomycin at 100 µg/mL (Corning). AT3 cells were cultured in complete RPMI-1640 media (Corning) containing 5% FBS, 2 mM L-glutamine, penicillin at 100 IU/mL plus streptomycin at 100 µg/mL and 1% HEPES (Corning). The PDX257S cell line [32,33] (established cell line from a primary xenograft BCa tumor) was cultured in cDMEM-10 media. Cell lines were authenticated by STR profiling (ATCC and Cellosaurus database search). All cells were grown in tissue-culture treated flasks (Corning) under standard sterile conditions (37 °C, 5% CO_2_) and cultured for at least two passages at ~75% confluency from any thawed cryopreserved vial prior to usage for in vivo challenge. Trypsin/EDTA solution (Corning) was used to digest and collect attached cells from flasks for cell counting by Trypan Blue (Invitrogen, Carlsbad, CA, USA) on a Countess Cell Counter (Invitrogen) prior to each passage.

### 2.4. Murine Tumor Models and Treatments

For the BBN model [34], male C56BL/6 or transgenic (CCL2^−/−^ or CCR2^−/−^) mice at 6–8 weeks of age were given drinking water, ad libitum, containing 0.05% BBN (TCI, Portland, OR, USA) which was prepared and replenished twice a week. After 4–5 months, bladders were harvested, weighed, and processed for immune profiling or preserved in 10% buffered formalin for subsequent hematoxylin and eosin (H&E) staining and histological diagnosis.

For the orthotopic bladder tumor model, as previously described [35], WT, transgenic (CCL2^−/−^, CCR2^−/−^, Rag1 KO), or bone marrow chimera female mice were anesthetized inside an isoflurane/O2 chamber with a heating pad to maintain optimal body temperature. The bladder from each animal was voided and instilled with a total of 100 µL of poly-L-lysine (MilliporeSigma, St. Louis, MO, USA) through a 24 G/0.56-inch urethral catheter (Becton Dickinson/BD Biosciences, Sandy, UT, USA) for 30 min (~50 µL of dead volume retained in catheter was included with a stopper closed at the end). The bladder was voided again and a total of 0.16 × 10^6^ MB49 cells in 100 µL of sterile phosphate-buffered saline (PBS) were instilled and retained for one hour. For the humanized model using NSG mice and a patient-derived BCa cell line created in our lab (PDX257s), 1 × 10^6^ PDX257S cells were instilled into the bladder under the same conditions [33]. Mice were numbered in order of instillation and distributed between control and treatment groups in an alternating pattern.

For subQ tumor models, as previously described [35], WT mice were challenged with 0.2 × 10^6^ MB49 cells in 100 µL of PBS subcutaneously on both flanks in the lower back of the animal. Tumor measurement was carried out by unblinded scientists using a caliper, and volume (mm^3^) was calculated by the formula:

$$volume=\frac{(length){\left(width\right)}^{2}}{2}$$

For the mammary fat pad challenge, 0.5 × 10^6^ AT-3 or MB49 cells in 50 µL of PBS with 50 µL of matrigel were injected into the 4th mammary fat pad on both sides. Tumors were measured by a caliper, and volume was calculated by the formula:

$$volume=\frac{(length){\left(width\right)}^{2}}{2}$$

For anti-CCL2 (αCCL2) treatment, anti-mouse CCL2 antibody (Clone 2H5, Bio X Cell, Lebanon, NH, USA) or isotype control antibody (Bio X cell) were injected intraperitoneally at 10 mg/kg per mouse in 100 µL PBS starting one day after any tumor challenge, twice a week for 3–4 weeks/until the end of study.

For T-cell adoptive transfer in the orthotopic model, murine splenocytes were harvested from WT (CD45.2 or CD45.1) or CCR2^−/−^ (CD45.2) female donor mice by crushing the spleens with the back of a 10 mL sterile syringe in 10 mL of RPMI-1640 media on a Petri dish and passing through 70 µm cell strainers. CD4^+^, CD8^+^, or total T cells were isolated using corresponding murine lymphocyte enrichment/negative-selection kits (BD Biosciences) and IMagnet (BD Biosciences). In total, 5 × 10^6^ of CD4^+^ or CD8^+^ T cells or 10 × 10^6^ total T cells were resuspended in 200 µL of sterile PBS and injected intravenously per CCR2^−/−^ (CD45.2) female recipient mouse through the tail vein using a 26G needle-attached 1 mL syringe one day before orthotopic challenge of MB49 cells. For NSG mice challenged with PDX257S cells, 7.5 × 10^6^ human peripheral blood mononuclear cells (PBMCs) were injected intravenously through the tail vein in 200 µL of sterile PBS per recipient prior to orthotopic challenge.

For gemcitabine treatment in the orthotopic model, 10 mg/mL of gemcitabine/GEMZAR (Lilly Medical) was instilled in 100 µL of PBS per mouse through a urethral catheter for one hour on days 1, 8, 15, and 22 post-orthotopic MB49 tumor challenge.

For recombinant CCL2 (rCCL2) treatment in the orthotopic model, a total of 800 ng of rCCL2 (human MCP-1 protein: R&D System, Minneapolis, MN, USA; mouse MCP-1 protein: BioLegend, San Diego, CA, USA) in 100 µL PBS was instilled per mouse through a urethral catheter for one hour on days 1, 8, 15, and 22 post-tumor challenge [16]. For treatment in the subQ model, 400 ng of rCCL2 (20 µg/kg) in 50 µL PBS was injected intratumorally starting at day 7 post-tumor challenge, every 5 days for 3 weeks.

For NK-cell depletion, WT or CCL2^−/−^ mice were injected intraperitoneally with 50 µL of anti-Asialo GM1 antibody (FUJIFILM Biosciences, Santa Ana, CA, USA) starting 4 days prior to tumor challenge, then weekly thereafter for 4 weeks. Mice in the control group were injected intraperitoneally with an equivalent amount of control irrelevant rabbit IgG (Jackson ImmunoResearch, West Grove, PA, USA).

For CD4^+^ or CD8^+^ T-cell depletion, WT or CCL2^−/−^ mice were injected intraperitoneally with 250 µg of anti-CD4 (Clone GK1.5, Bio X Cell), anti-CD8 (Clone YTS 169.4, Bio X Cell), or isotype control monoclonal antibody (mAb; Bio X Cell) in 100 µL of PBS starting one day before orthotopic tumor challenge and twice a week thereafter.

A humane endpoint was applied, and animals were euthanized immediately whenever any of these criteria were met: (1) any subQ or mammary fat pad tumor reached over 2000 mm^3^; (2) any severe hematuria resulted from orthotopic implantation of tumor cells accompanied by worsened body condition (weight loss > 20%, low body temperature, reduced mobility or intake of food/water, etc.); (3) end of the experiment. The bodily conditions of the mice were monitored daily, with hematuria checked weekly using Chemstrip 7 urine strips (Roche Diagnostics, Indianapolis, IN, USA).

### 2.5. Construction of Bone Marrow Chimeric Mice

Drinking water was supplemented with 1 mg/mL of neomycin (Alfa Aesar/Thermo Scientific Chemical, Waltham, MA, USA) and given to female WT (CD45.2) recipient mice at 6–10 weeks of age starting 3–5 days prior to irradiationand bone marrow transfer then continuously for approximately two weeks. Antibiotics-supplemented drinking water was prepared fresh and replaced every 3–4 days. Recipient mice received 900 rads of X-ray irradiation. On the same day, 6-week-old donor female mice (WT/CD45.1 and CCR2^−/−^/CD45.2) were sacrificed and femurs and tibias were removed to collect bone marrow by cutting both ends of the bones and flushing out with cold, serum-free RPMI-1640 using a 25–26 G needle-attached syringe. Bone marrow clumps were broken up by passing through a 21 G needle and spun down by centrifugation at ~300 rcf/g for 7 min. The cell pellet was loosened by gently tapping the tube after decanting the supernatant and red blood cells were removed using hypotonic shock by adding 9 mL of sterile water for 5–7 s then 1 mL of sterile 10× PBS. Cell suspension was passed through a 70 µm cell strainer to remove any debris and counted by a cell counter (Vi-Cell, Beckman Coulter Life Sciences, Indianapolis, IN, USA) prior to staining with PE anti-mouse CD4 (Clone GK1.5; BioLegend) and CD8 mAbs (Clone 53-6.7; BioLegend) and depletion of both populations by EasySep PE-positive selection (STEMCELL technologies, Vancouver, BC, Canada). WT/CD45.1 and CCR2^−/−^/CD45.2 bone marrow cells (no red blood cells or mature CD4^+^ and CD8^+^ T cells) were mixed at a 1:1 ratio and 4 × 10^6^ total donor cells were injected intravenously per recipient/CD45.2 mouse through the tail vein using a 26 G needle-attached 1 mL syringe. Bone marrow chimeric mice were carefully monitored and switched from antibiotic to normal drinking water approximately two weeks after adoptive transfer/irradiation when they were stable with no signs of internal infection or any complications. Reconstitution of a mixture of CD45.1/CD45.2 immune populations was confirmed by flow cytometry staining and analysis on a small amount of cheek blood taken from each chimera mouse approximately 4 weeks after adoptive transfer of donor bone marrow cells prior to orthotopic challenge of MB49 cells.

### 2.6. Immune Analysis by Flow Cytometry

Murine bladders were harvested around ~week 3 post-orthotopic MB49 challenge or the end of the study of the BBN model. As previously described [32], bladders were minced and digested in 3 mL of RPMI-1640 per 0.5 g of tissue with 1.5 mg/mL of collagenase IV (MilliporeSigma) and 0.15 mg/mL of DNase I (MilliporeSigma) for 45 min at 37 °C, 5% CO_2_. Digestion was stopped by adding 2× volume of complete RPMI (cR-10) media (10% FBS, 2 mM L-glutamine, 100 IU/mL penicillin, and 100 µg/mL streptomycin) and passing through 100 µm cell strainers to obtain a single-cell suspension (SCS) for staining.

In mixed bone marrow chimeric mice challenged with MB49 orthotopically, spleen and tumor-draining/retroperitoneal lymph nodes were also collected and passed through 100 µm cell strainers to obtain SCSs for staining.

For intracellular cytokine detection later, cells were first resuspended in cR-10 media at up to 1 × 10^6^ cells/100 µL per well on a 96-well U-bottom plate and incubated with Cell Activation Cocktail containing Brefeldin A (BioLegend) for 5 h at 37 °C, 5% CO_2_.

For cell surface staining, murine or human SCS samples were then washed and resuspended in cold flow buffer (2% FBS in PBS) at up to 1 × 10^6^ cells/100 µL on a 96-well U-bottom plate, blocked with mouse Fc-blocker for 10 min at 4 °C, followed by incubation with a mixture of fluorochrome-conjugated antibodies at ~0.1–0.2 µg each (titrated optimal binding/detection) for 45 min at 4 °C. SCSs were washed with flow buffer again and either fixed with 2% paraformaldehyde or proceeded to intracellular staining using Cytofix/Cytoperm™ Fixation/Permeabilization Kit (BD Biosciences) and 0.2–0.3 µg of fluorochrome-conjugated antibodies.

Samples were run using the LSR II FACSDiva software version 8.0.1 (BD Biosciences) and analyzed using FlowJo software version 10.10.0 (FlowJo). Compensation was performed with single-color controls prepared using BD Biosciences CompBeads. Compensation matrices were calculated automatically, and sample analysis was carried out using FACSDiva software or FlowJo software. The antibodies and dyes used are as follows: Fixable Viability Dye eFluor™ 455UV (Invitrogen); fluorochrome-conjugated anti-human antibodies for markers—CD45 (Clone HI30, BioLegend), CD3 (Clone SK7, BioLegend), CD25 (Clone BC96, BioLegend), CD69 (Clone FN50, BioLegend), CD107a (Clone H4A3, BioLegend), CD56 (NCAM) (Clone 5.1H11, BioLegend), CD3 (Clone UCHT1, BioLegend), CD4 (Clone OKT4, BioLegend), CD8 (Clone SK1, BioLegend), CD14 (Clone HCD14, BioLegend), IFNγ (Clone 4S.B3, BioLegend), TNF-α (Clone Mab11, BioLegend), granzyme B (Clone QA16A02, BioLegend) and perforin (Clone dG9, BioLegend); fluorochrome-conjugated anti-mouse antibodies for markers—CD45 (Clone 30-F11, BioLegend), CD11b (Clone: M1/70, BioLegend), Gr-1 (Clone RB6-8C5, BioLegend), Ly-6G (Clone 1A8, BioLegend), Ly-6C (Clone HK1.4, BioLegend), CD11c (Clone N418, BioLegend), F4/80 (Clone BM8, BioLegend), CD206 (MMR) (Clone C068C2, BioLegend), CD3 (Clone 17A2, BioLegend), CD4 (Clone GK1.5, BioLegend), CD8a (Clone 53-6.7, BioLegend), TCR γ/δ (Clone GL3, BioLegend), CD69 (Clone H1.2F3, BioLegend), NK-1.1 (Clone PK136, BioLegend), IFNγ (Clone XMG1.2, BioLegend), TNF-α (Clone MP6-XT22, BioLegend), granzyme B (Clone QA16A02, BioLegend), perforin (Clone S16009A, BioLegend), CCR2 (Clone SA203G11, BioLegend). Cell-sorting experiments were carried out on FACS Cell Sorter Aria or Symphony S6 (BD).

### 2.7. Histopathology Examination of Human CCL2 Staining

Human bladder tumor tissues were fixed in 10% buffered formalin and then paraffin-embedded for tissue sectioning [33]. Slides were stained with an anti-human CCL2 antibody (GeneTex, Irvine, CA, USA; Cat. No. GTX37379, 1:200–1:50). All sections were assessed by pathologists who were blinded to group assignments. To account for patient-to-patient variability, CCL2 staining was calculated with internal normalization as follows: the staining evaluation was based on the intensity (weak = 1, moderate = 2, and high = 3) of CCL2 staining and the density (0% = 0, 1–40% = 1, 41–75% = 2, >76% = 3) of positive cells. The final score for each tumor section sample was calculated by multiplying the intensity and density scores. Control staining scores were obtained from the lamina propria section of each sample. The tumor vs. CCL2 ratio was classified as high (>1) vs. low (≤1) based on whether the tumor CCL2 score was higher than the interphase CCL2 score.

### 2.8. Human Bladder Tissue Processing and Storage

As previously described [36], bladder tissues (tumor or adjacent normal) were surgically excised under sterile conditions and placed in Transport Media containing RPMI-1640, 2% FBS, 25 mM HEPES, penicillin at 100 IU/mL, and streptomycin at 100 µg/mL and transported on ice. Fresh tumor tissues were washed with PBS and minced into 1–2 mm pieces and incubated with 5 mL of 0.05% trypsin/EDTA per 1 g of tissue supplemented with 1 mg/mL of collagenase IV (MilliporeSigma) and 0.25 mg/mL of DNase I (MilliporeSigma) for 45 min at 37 °C, 5% CO_2_ with shaking. The digestion was then stopped by adding 2× the volume of cR-10 media and the samples were filtered through a 100 µm cell strainer to obtain SCSs for cryopreservation in freezing media containing RPMI-1640, 50% FBS, and 10% DMSO and stored at −150 °C until use.

### 2.9. Human PBMC and BCa Tumor Draining Lymph Node (TDLN) Processing and Storage

As previously described [32], clinical blood samples were collected in BD Vacutainer Heparin Tubes (BD), and PBMCs were isolated from plasma-removed whole blood using Ficoll gradient media (GE Healthcare, Chicago, IL, USA) and cryopreserved in freezing media as mentioned above and stored at −150 °C until use. Fresh TDLNs were collected during cystectomy and transported on ice in Transport Media, crushed in 5 mL of cR-10 media with the back of a sterile 10 mL syringe, and then filtered through a 100 µm cell strainer to obtain SCSs for cryopreservation as mentioned above.

### 2.10. Transwell Migration Assay

Human PBMCs were thawed using 1× Anti-Aggregate Wash (Cellular Technology Limited/CTL, Shaker Heights, OH, USA) prepared in RPMI-1640 media and resuspended in cDMEM-10 media at 10 × 10^6^ cells/mL after centrifugation. In total, 100 µL of PBMCs (1 × 10^6^ total) was added to each top insert of the Transwell plate (6.5 mm/5.0 µm; 24-well plate; Corning) pre-wetted with media [37]. Media alone or with human rCCL2 (H.rCCL2) was added to the bottom chambers at 100 ng/mL in 600 µL of cDMEM-10 per well of Transwell plate, with pre-incubation of 10 min at RT with or without 2 µg/mL of isotype control goat antibody (R&D System) or goat anti-human CCL2 antibody (R&D System), or addition of CCR2 antagonist (RS 504393; TOCRIS-BioTechne, Minneapolis, MN, USA) at 10 µM. Top inserts with PBMCs were placed carefully into each well/bottom chamber and incubated at 37 °C for 2 h. After removing the top insert, content from the bottom chamber was collected into 1.5 mL microcentrifuge tubes and migrated cells were spun down by centrifugation followed by flow cytometry staining and analysis.

### 2.11. Detection of CCL2 Expression in BCa

The presence of human CCL2 in the urine of healthy individuals or patients with BCa (with or without tobacco use) was measured by ELISA (PeproTech, Cranbury, NJ, USA) [38]. BCa tumor SCSs or adjacent normal tissue SCSs were thawed and resuspended in cDMEM-10 media at 0.5 × 10^6^ cells/mL and incubated at 37 °C, 5% CO_2_ for 48 h, then the supernatant was collected for human CCL2 ELISA (PeproTech). Normal bladders from naïve mice or bladders with tumors from the MB49 orthotopic model or BBN model were processed into SCSs and cultured in cR-10 media at 0.5 × 10^6^ cells/mL, 50 µL per well on a 96-well plate and incubated at 37 °C, 5% CO_2_ for 24 h. Supernatants were collected and measured by a mouse chemokine array (R&D System) [39] or murine CCL2 ELISA (R&D System) [40].

### 2.12. In Vitro Culture of rCCL2 with Cells and Subsequent Analysis

Human PBMCs, TDLNs, and BCa tumor SCSs were thawed using Anti-Aggregate Wash to maximize viability and recovery prior to resuspension in cR-10 media and cell counting. PBMCs were resuspended in cR-10 media at ~0.5 × 10^6^ cells/mL on a 96-well flat-bottom plate with or without the presence of H.rCCL2 at 800 ng/mL for 24 h before proceeding to flow cytometry staining for activation markers (CD25 and CD69).

For tumor-infiltrating CD4^+^ and CD8^+^ conventional T cells (Tconv), BCa tumor SCSs from the Ta/T1 stage were first stained with FVD and flow cytometry antibodies. Tumor-infiltrating Tconv cells were then sorted by FACS and cultured in cR-10 media at 0.2 × 10^6^ cells/well on a 96-well flat-bottom plate with or without 800 ng/mL of H.rCCL2 for 24 h and then analyzed by flow cytometry for expression of activation markers.

For total RNAseq analysis [41], matching PBMCs and TDLNs were pooled from the same group of patients with Ta/T1 BCa tumors. The samples were first stained with FVD and flow cytometry antibodies. Live peripheral vs. TDLN CD4^+^ and CD8^+^ Tconv were sorted by FACS, then cultured at 0.5 × 10^6^ cells/well in cR-10 media on a 96-well flat-bottom plate with or without 800 ng/mL of H.rCCL2 for 24 h prior to RNA extraction (QIAGEN, Germantown, MD, USA) and gene expression analysis (Novogene, Sacramento, CA, USA). RNA was extracted using the RNeasy Mini or Micro Kit (QIAGEN) according to the manufacturer’s suggested protocol. RNA samples were frozen and stored at −80 °C until the time of shipment to Novogene Corporation, Inc. Construction, and sequencing of the cDNA library were performed using the Illumina NovaSeq 6000 utilizing a short-read strategy. Sample data quality was guaranteed (>93% bases with Q30 or >97% with Q20). Differential gene expression quantification analysis was carried out with bioinformatic approaches as follows by Novogene: (1) the overall results of the expected number of Fragments Per Kilobase of transcript sequence per Millions of base pairs sequenced (FPKM) analysis in the form of heatmap was plotted using the log2(FPKM + 1) value of a list of genes related to T-cell activation/function; (2) the fold change in gene expression between different samples was visualized in the form of volcano plots of lists of genes related to T-cell chemokines/cytokines or cell surface markers.

Human BCa T24 cells or mouse MB49 cells were cultured in cDMEM-10 media at ~0.5 × 10^6^ cells/mL on a 96-well flat-bottom plate with or without the presence of H.rCCL2 or mouse rCCL2 at 800 ng/mL for 24 h. The viability of cells was measured by flow cytometry analysis after digestion of attached cells with trypsin/EDTA for ~30 s and staining with FVD.

### 2.13. Human IFNγ ELISPOT

As previously described [42], using Human IFNγ ELISPOT Set (BD), anti-human IFNγ capture mAbs were coated on ELISOT plates overnight at 4 °C, then blocked with 1% bovine serum albumin (BSA, MilliporeSigma) in sterile PBS after wash. TDLN SCSs were thawed using Anti-Aggregate Wash and resuspended in cR-10 media at ~0.5 × 10^6^ cells/100 µL per well, followed by mixing with another 100 µL of cR-10 media, or containing 1600 ng of H.rCCL2, with or without 0.25 µg of anti-human HLA-ABC mAb (Clone W6/32, BioLegend) and anti-human HLA-DR (Clone L243, BioLegend) mAb. All conditions were performed in duplicate, triplicate, or quadruplicate for any independent assay. Plates were incubated at 37 °C with 5% CO_2_ for 24 h. Afterward, wells were incubated with anti-human IFNγ detection mAb at 4 °C overnight after washes. Plates were then incubated with streptavidin-HRP at room temperature for 1.5 h, followed by development with 3-Amino-9-Ethylcarbazole (AEC) substrate. The reaction was stopped by deionized water after spots were formed. IFNγ-producing spot-forming cells (SFCs) were quantified using an ImmunoSpot analyzer (S6 Micro, CTL) with ImmunoSpot software, version 6.0 (CTL).

### 2.14. Bioinformatics

The Cancer Genome Atlas (TCGA) database was used for this approach. From this database, the gene expression and survival data for 212 BCa patients was gathered [43] which includes *n* = 100 patients with MHC low tumors and *n* = 112 patients with MHC high tumors.

### 2.15. Statistics

To assess bladder weights and individual immune subpopulations between any two groups in all mouse studies, an unpaired *t*-test was performed. Differences among three or more treatment groups were assessed using a one-way ANOVA with Tukey’s multiple comparisons test. For non-normal data (*p* < 0.05 by D’Agostino & Pearson test), non-parametric tests (Mann–Whitney or Kruskal–Wallis, respectively) were performed. For normal data with unequal standard deviations (*p* < 0.05 by F test or Brown–Forsythe test, respectively), a Welch’s *t*-test or Brown–Forsythe and Welch ANOVA with Dunnett’s T3 multiple comparisons test was performed. To evaluate the incidence of carcinogenesis between two groups in mouse studies, Fisher’s exact test was utilized. For analyzing tumor growth differences between two groups in mouse studies, two-way ANOVA was employed. In survival studies, the log-rank/Mantel–Cox test was applied to compare survival differences between two groups as depicted in Kaplan–Meier plots. For multiple comparisons between survival curves, Holm-Šídák’s multiple comparisons test was performed. Using pilot experiment data from our orthotopic model to calculate the effect size, an a priori power analysis was performed to determine an appropriate sample size to achieve 80% power with an error probability of 0.05. For experiments with no pilot data, a post hoc power analysis was performed to confirm appropriate power was achieved based on the sample size. Analyses were performed using Prism v11. For in vitro assays involving human PBMCs, TDLNs, or sorted bladder tumor-infiltrating T cells based on either triplicated data from representative patients or pooled data from at least three patients, paired *t*-tests were carried out to test the differences between tumor and matched adjacent normal tissue. Volcano plot analysis was employed to investigate significant differential gene expression fold changes between control and treatment groups. Additionally, Spearman correlation analysis was performed to examine the relationship between specific gene expressions in the relevant patient cohorts.

## 3. Results

### 3.1. The CCL2/CCR2 Axis Exerts Anti-Tumor Effects in Mouse BCa

**Figure 1:**
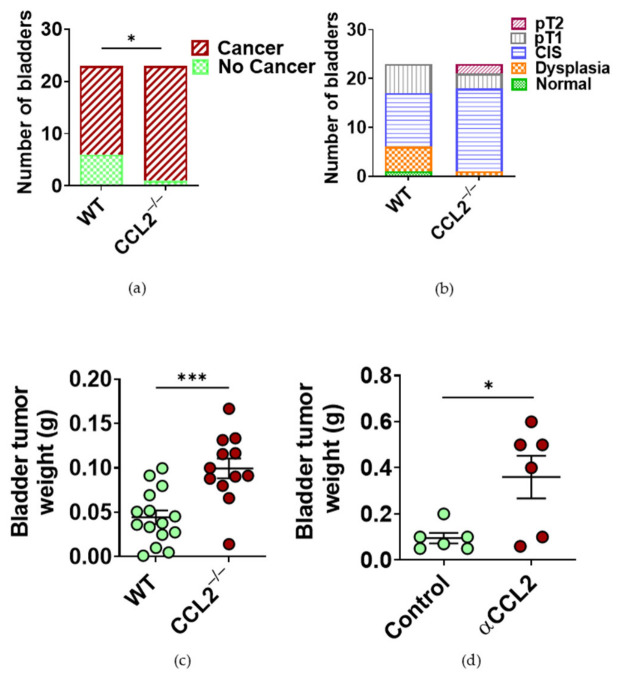
The CCL2/CCR2 axis exerts anti-tumor effects in different murine models of BCa. WT C57BL/6 (WT; *n* = 23) and CCL2 KO (CCL2^−/−^; *n* = 23) mice were given BBN in drinking water for 4.5 months to induce bladder carcinogenesis. Mice were sacrificed and bladders were processed for histopathologic examination. (**a**) Carcinogenesis was measured by the pathological incidences. Dysplasia/normal—no cancer; CIS/pT1/pT2—cancer. *p*—Fisher’s exact. (**b**) Urothelium was classified as normal urothelium, urothelial dysplasia, carcinoma in situ (CIS), pT1 tumor (tumor has spread to the lamina propria), or pT2 tumor (tumor has invaded the muscle). (**c**) WT (*n* = 15) and CCL2^−/−^ (*n* = 12) mice were challenged orthotopically with 8 × 10^4^ MB49 cells and sacrificed after ~3 weeks for the determination of bladder weights. Shown are the pooled bladder weights from two independent experiments. Mean ± SEM, *** *p* ≤ 0.001, unpaired *t*-test. (**d**) WT mice were challenged orthotopically with 8 × 10^4^ MB49 cells and treated intraperitoneally with anti-CCL2 (αCCL2; *n* = 6) 10 mg/kg, starting from day 1 after tumor challenge, twice a week. Mice were sacrificed after ~3 weeks for the determination of bladder weights compared to control (*n* = 6). Mean ± SEM, * *p* ≤ 0.05, Welch’s *t*-test.

Previous work has found that myeloid cells are one of the most abundant tumor-infiltrating immune cells in BCa [44], and that intravesical chemotherapy boosted immune therapy in the bladder by modulating tumor macrophages [45]. Based on these findings and published data on CCL2 in other tumors [20,21,22,46,47,48], we hypothesized that CCL2-mediated recruitment of myeloid cells promotes BCa development and that CCL2 could be targeted to treat BCa. To investigate, the BBN chemically-induced BCa model was used [49]. BBN, a metabolite of *N*-nitroso-di-*N*-butylamine, is found in tobacco smoke and specifically induces bladder carcinogenesis in rodents that mimics the histology of human BCa [50]. Male mice were administered BBN in their drinking water which spontaneously induced urothelial cancer after 4 months. Unexpectedly, we found that BBN-induced BCa was more prevalent in CCL2^−/−^ mice compared to WT mice (Figure 1a), and some CCL2^−/−^ mice had a higher tumor stage (i.e., ≥T2) compared to WT mice (Figure 1b). To further examine the role of CCL2 in BCa growth, a syngeneic orthotopic model of MB49 mouse BCa was used. MB49 tumor growth, measured by bladder weights, was increased in CCL2^−/−^ compared to WT mice (Figure 1c). Combined, these data suggest that CCL2 protects against carcinogenesis and tumor growth in the bladder.

Based on the pro-tumorigenic function of CCL2 in other cancers [19], αCCL2 antibodies have been used to treat some cancers, including breast, and have undergone human testing [27]. However, our findings suggest that αCCL2 may be harmful to patients with BCa. To determine the effects of blocking CCL2 across tumor types, we tested αCCL2 in the treatment of MB49 BCa versus AT-3 mammary tumors. As previously published [27], we confirmed that αCCL2 decreased AT-3 growth in orthotopic mammary fat pads (Supplementary Figure S1A). However, blocking CCL2 signaling with αCCL2 accelerated the growth of MB49 BCa in the bladder environment (Figure 1d). Further, when heterotopically transplanted into the mammary fat pads, MB49 bladder tumors responded to αCCL2 therapy similar to AT-3 tumors (Supplementary Figure S1B). These findings demonstrate that the anti-tumor effects associated with CCL2 are environment-specific, supporting a tissue-specific, anti-tumor role of CCL2 in the bladder environment compared to its established tumor-promoting role in the mammary environment.

### 3.2. Anti-Tumor Effects of CCL2/CCR2 in the Bladder Require T Cells

**Figure 2:**
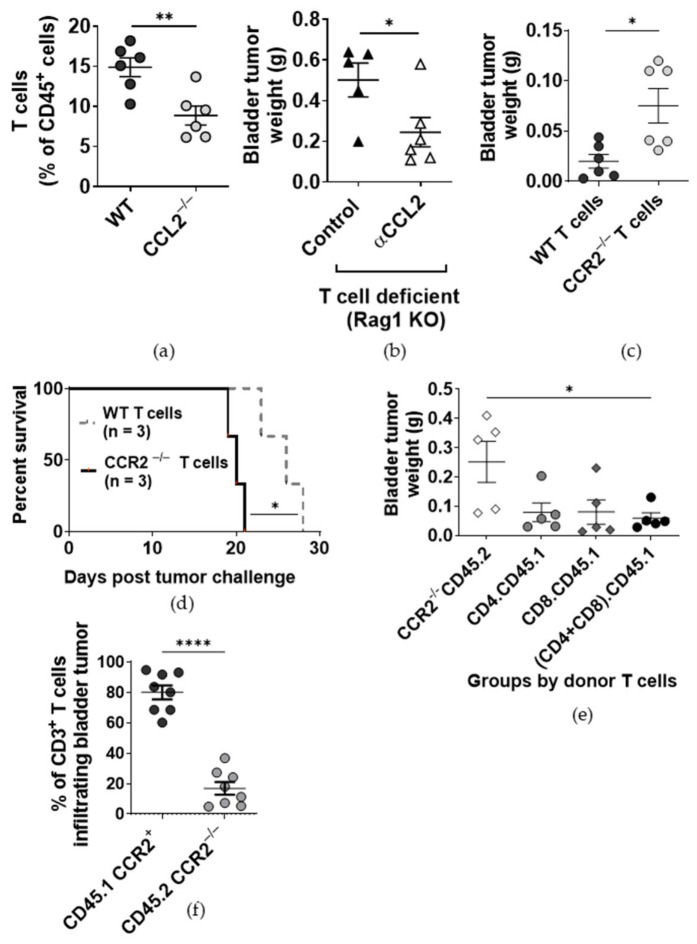
CCL2/CCR2 protects against BCa in a T-cell-dependent manner. WT C57BL/6 and CCL2^−/−^ mice were challenged orthotopically with 8 × 10^4^ MB49 cells and sacrificed after ~3 weeks for bladder tumor processing. (**a**) Bladder tumor-infiltrating T cells (CD45^+^CD3^+^) were detected by flow cytometry. (**b**) Rag1 KO mice were challenged orthotopically with 8 × 10^4^ MB49 cells and treated intraperitoneally with anti-CCL2 (αCCL2) 10mg/kg, starting from day 1 after tumor challenge, twice a week. Mice were sacrificed after ~2 weeks for the determination of bladder weights. (**c**,**d**) CD3^+^ T cells (10^−12^ × 10^6^) were isolated magnetically from WT or CCR2^−/−^ mice, and then adoptively transferred via intravenous injections into CCR2^−/−^ recipients. One day after adoptive transfer, mice were challenged orthotopically with 8 × 10^4^ MB49 cells. (**c**) In one cohort, mice were sacrificed after ~3 weeks for the determination of bladder weights. Mean ± SEM, * *p* ≤ 0.05, ** *p* < 0.01, unpaired *t*-test. (**d**) In another cohort, mice were followed for survival Kaplan–Meier plots comparing survival in the aforementioned groups. * *p* ≤ 0.05, log-rank (Mantel–Cox) test. (**e**) CD4 and CD8 were isolated magnetically from CD45.1 WT mice and then adoptively transferred via intravenous injections into CD45.2 CCR2^−/−^ mice. One day after adoptive transfer, mice were challenged orthotopically with 8 × 10^4^ MB49 cells. Mice were sacrificed after ~3 weeks for the determination of bladder weights. Mean ± SEM, * *p* ≤ 0.05 by one-way ANOVA with Tukey’s multiple comparisons test. (**f**) CD45.1 CCR2^+^/CD45.2 CCR2^−/−^ bone marrow chimeric mice were challenged orthotopically as described in the Materials and Methods and sacrificed after ~3 weeks. Bladders were harvested and processed for immune analysis by flow cytometry of immune infiltrates. Mean ± SEM, * *p* ≤ 0.05, **** *p* < 0.0001, unpaired *t*-test.

CCL2 recruits and activates multiple types of lymphocytes during inflammation [51]. To identify lymphocytes potentially responsible for CCL2-mediated protection, we analyzed bladder-infiltrating lymphocytes in WT vs. CCL2^−/−^ mice challenged with MB49 bladder tumors. WT mice had significantly more intratumoral T cells (Figure 2a) compared to CCL2^−/−^ mice among lymphocytes. To determine if T cells contribute to the protective role of CCL2, we tested the growth of MB49 bladder tumors in WT vs. CCL2^−/−^ during CD4 or CD8 T-cell depletion. Depletion of either CD4^+^ or CD8^+^ T cells abolished the protective effect of CCL2 (Supplementary Figure S2). To confirm that the suppression of tumor growth by CCL2 is dependent on T cells, we examined the impact of αCCL2 antibody in WT versus T-cell-deficient Rag1 KO mice and found that αCCL2 did not promote tumor growth in T-cell-deficient Rag1 KO (Figure 2b). Moreover, in the absence of T cells, control mice had accelerated tumor growth compared to mice treated with αCCL2 antibody (Figure 2b), indicating competing roles of CCL2 in protecting against tumor growth through T cells and promoting BCa growth through other non-T-cell mechanisms. These findings support the dual role of CCL2 in tumor-protective and tumor-promoting properties in the bladder environment. However, protection against tumor growth appears to dominate in the bladder, resulting in an overall protective outcome facilitated by CCL2.

The primary chemokine receptor for CCL2 is CCR2. In the BBN carcinogenesis model, a significant increase in bladder weights was observed in CCR2^−/−^ compared to WT mice (Supplementary Figure S3A) accompanied by a non-significant increase in cancer incidence (Supplementary Figure S3B,C), supporting the role of CCR2 in BCa carcinogenesis. To determine if CCR2 protection was also T-cell-dependent, we adoptively transferred WT (CCR2^+^) versus CCR2^−/−^ T cells into CCR2-deficient (CCR2^−/−^) mice challenged with MB49. Compared to mice receiving CCR2^−/−^ T cells, mice receiving CCR2-replete (WT) T cells were protected from BCa growth, marked by decreased bladder weights (Figure 2c) and extended survival (Figure 2d). Adoptive transfer of both WT CD4^+^ and CD8^+^ T cells was required to reduce bladder weights in CCR2^−/−^ mice challenged with MB49 (Figure 2e), supporting the need for both CD4 and CD8 T-cell subsets in the protective effect of CCR2. To further confirm that CCR2-dependent T-cell function is important for T-cell control of BCa, we generated mixed bone marrow chimeric mice in which lethally irradiated mice are reconstituted with a 1:1 mix of wild-type bone marrow (CD45.1) and bone marrow from CCR2-deficient mice (CD45.2) and subsequently challenged with MB49 tumor cells. CD45.1 CCR2^+^ T cells were preferentially recruited to the bladder compared to CD45.2 CCR2^−/−^ T cells (Figure 2f); this ratio was reversed in the spleen and lymph node (Supplementary Figure S4A,B). Further, we found that CD45.1 CCR2^+^ T cells were more activated (CD25^+^) and tumor-specific (tetramer^+^) and expressed significantly higher IFNγ, granzyme B, and perforin compared to CD45.2 CCR2^−/−^ T cells in bladder tumors (Supplementary Figure S5A–E).

### 3.3. Intravesical Recombinant CCL2 Instillation Treats BCa

**Figure 3:**
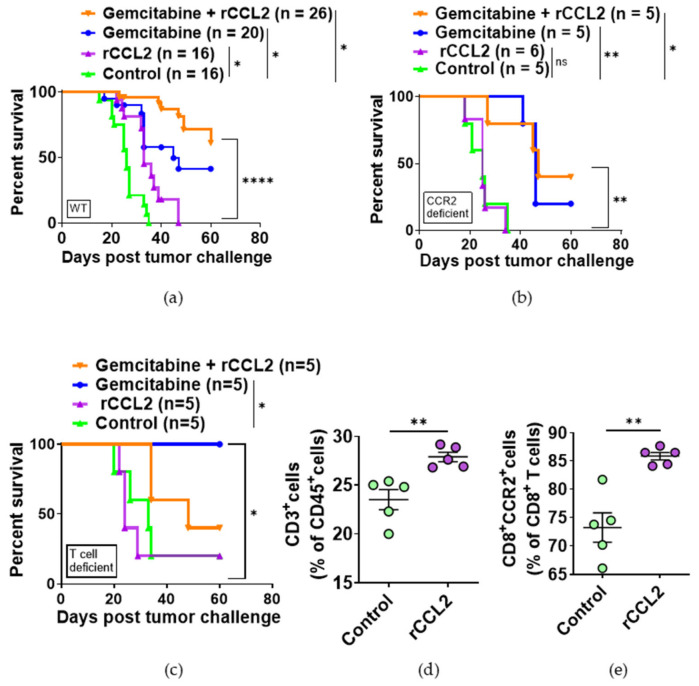

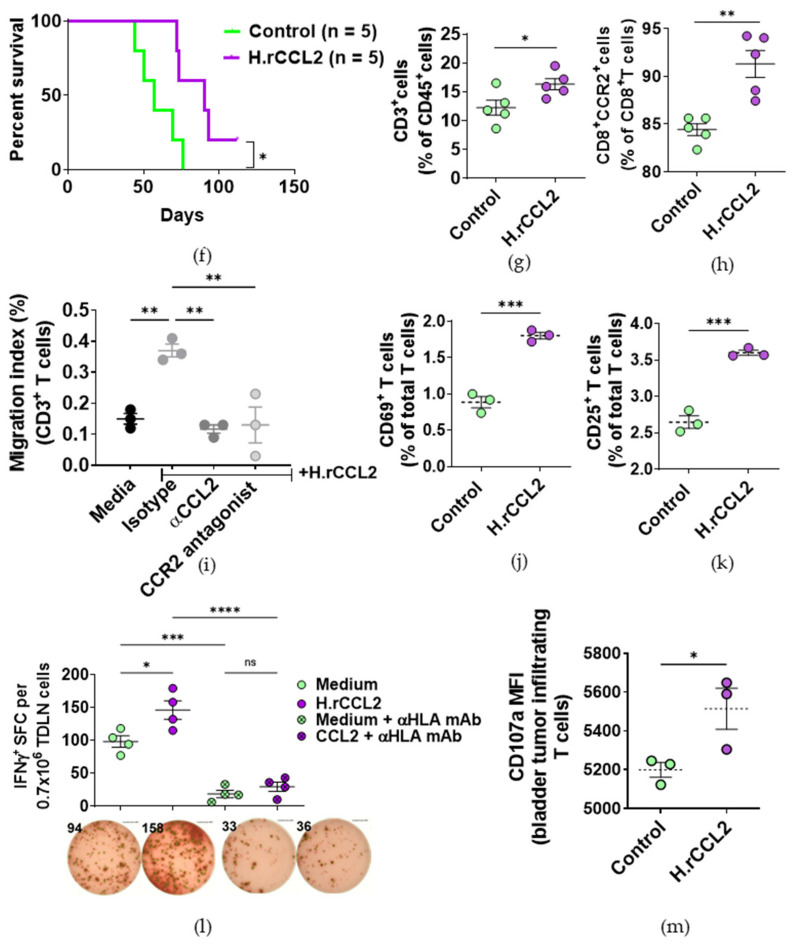
Recombinant CCL2 shows T-cell-mediated therapeutic efficacy in BCa. B6 WT (**a**), CCR2^−/−^ (**b**), and TCRα KO (**c**) female mice were challenged orthotopically with 8 × 10^4^ MB49 BCa cells and were divided into four groups: control PBS, rCCL2 only (intravesically instilled with 800 ng/100 μL rCCL2 starting from day 1 and repeated weekly), gemcitabine only (intravesically instilled with gemcitabine at 10 mg/mL starting at day 1 and repeated weekly), gemcitabine + rCCL2 (intravesically instilled with 800 ng/100 μL rCCL2 plus gemcitabine 10 mg/mL, started at day 1 and repeated weekly). Kaplan–Meier plots comparing survival in the aforementioned groups. * *p* ≤ 0.05, ** *p* < 0.01, *** *p* < 0.001, **** *p* < 0.0001, log-rank (Mantel–Cox) test with Holm-Šídák’s multiple comparisons test. Female B6 mice were challenged orthotopically with 8 × 10^4^ MB49 BCa cells and were intravesically treated with rCCL2 only (800 ng/100 μL rCCL2 starting from day 1 and repeated weekly). Mice were sacrificed at ~3 weeks, bladders were processed and (**d**) CD3^+^ T cells and (**e**) CCR2^+^CD8^+^ T cells were detected by flow cytometry. Mean ± SEM; ** *p* ≤ 0.01, unpaired *t*-test (**d**) or Welch’s *t*-test (**e**). (**f**) 7.5 × 10^6^ PBMCs were given via tail vein injections to female WT mice who were orthotopically challenged the next day with 1.2 × 10^6^ PDX257S bladder tumor cells. From day 1, human CCL2 (H.rCCL2) was instilled intravesically (800 ng in 100 μL catheter injection volume) weekly for 4 weeks. (**f**) In one cohort, survival is followed. Kaplan–Meier plots were used to compare survival. * *p* ≤ 0.05, log-rank (Mantel–Cox) test. In another cohort, mice were sacrificed, bladders were processed and (**g**) CD3^+^ T cells and (**h**) CCR2^+^CD8^+^ T cells were detected by flow cytometry. Mean ± SEM; * *p* ≤ 0.05, ** *p* ≤ 0.01, unpaired *t*-test. (**i**) Human PBMCs were placed in the top insert of a Transwell plate system, while the media in the bottom chamber contained anti-CCL2 (αCCL2) antibody, isotype control antibody, or CCR2 antagonist. After 2 h, content in the bottom chamber was collected and the absolute number (AN) of migrated CD3^+^ T cells was analyzed by flow cytometry. Migration index = AN of migrated cells/AN of non-migrated cells. Shown are the pooled results of three independent experiments and a representative from at least 3 patients with BCa. Mean ± SEM; ** *p* < 0.01, one-way ANOVA with Tukey’s multiple comparisons test. (**j**,**k**) PBMCs were co-cultured with 800 ng/mL of CCL2 with medium only as the control in triplicate for 24 h then analyzed by flow cytometry for expression of activation markers such as (**j**) CD69 and (**k**) CD25 in the live CD45^+^CD3^+^ T-cell population. Shown are the representative results of at least 3 patients; * *p* ≤ 0.05, unpaired *t*-test. (**l**) TDLNs from patients were measured for IFNγ spot-forming cells (SFCs) by ELISPOT assay with or without the presence of anti-human HLA-A, B, C, and DR mAbs to block Ag-presentation to T cells as described in the Materials and Methods. Shown are representative results in triplicate from 1 out of 3 patients. The bottom images are one example of an ELISPOT well from replicates of each condition. Mean ± SEM; * *p* < 0.05, ** *p* < 0.01, *** *p* < 0.001, **** *p* < 0.0001, ns; not significant, one-way ANOVA with Tukey’s multiple comparisons test. (**m**) As described in the Materials and Methods, tumor-infiltrating CD4^+^ and CD8^+^ T cells were sorted, then co-cultured with 800 ng/mL of H.rCCL2 or with medium only as a control in triplicate for 24 h then analyzed by flow cytometry for expression of activation markers, including the degranulation marker, CD107a, in the live CD45^+^CD3^+^ T-cell population. Shown are the pooled results of at least 3 patients with Ta or T1 BCa.

Given that CCL2 is protective in the bladder environment, we hypothesized that recombinant CCL2 (rCCL2) could be delivered into the bladder to treat BCa. Intravesical administration of chemotherapy is a common approach to treating non-muscle invasive BCa. For example, intravesical gemcitabine significantly decreases the risk of tumor relapse in patients with non-muscle invasive BCa following tumor resection [52] and is currently the standard-of-care agent given immediately after tumor resection. We delivered intravesical rCCL2 with or without intravesical gemcitabine to mice challenged with MB49 tumors and measured survival relative to the control group. Intravesical rCCL2 was effective as a single agent and in combination with gemcitabine as shown by significant improvement in survival over control (Figure 3a). Combination treatment was highly effective as 90% of mice receiving rCCL2 and gemcitabine survived as late as 60 days after tumor inoculation (Figure 3a). We confirmed that the effectiveness of rCCL2 required both CCR2 and T cells as the treatment was not effective in CCR2^−/−^ mice (Figure 3b) nor in mice lacking T cells (Figure 3c). Treatment of MB49 bladder tumors with rCCL2 increases the frequency of bladder tumor-infiltrating CD3^+^ total T cells and CCR2^+^CD8^+^ T cells (Figure 3d,e).Further, rCCL2, given intratumorally, significantly reduced MB49 subcutaneous tumor growth (Supplementary Figure S6).

### 3.4. Relevance of the Anti-Tumor CCL2/CCR2 Axis in Human BCa

Next, Human Recombinant CCL2 (H.rCCL2) was tested using a double humanized model of BCa where NSG mice were engrafted with patient-derived BCa cells and human PBMCs to simulate the tumor–immune microenvironment. Compared to the placebo control, H.rCCL2 increased the survival of PDX tumor-bearing mice (Figure 3f) and increased the frequency of human CD3^+^ T cells and CCR2^+^CD8^+^ T cells in mouse bladder tumors (Figure 3g,h). To identify specific H.rCCL2-mediated effects on patient-derived T cells in BCa, we tested the effects of H.rCCL2 on the migration ability of patient-derived peripheral blood T cells and found that H.rCCL2 significantly improved the migration of these T cells in vitro and this migration was inhibited by αCCL2 blocking antibodies and a CCR2 antagonist (Figure 3i), highlighting the specific involvement of the CCL2/CCR2 axis in the H.rCCL2-mediated migration of human T cells. Additionally, H.rCCL2 increased the frequency of activated CD69^+^ (Figure 3j) and CD25^+^ patient-derived peripheral blood T cells in vitro (Figure 3k). RNA sequencing of H.rCCL2-treated T cells derived from patient TDLNs showed that H.rCCL2 increased the gene expression of cytokines, their receptors, and T-cell activation markers (Supplementary Figure S7). The ELISPOT assay revealed that H.rCCL2 treatment also increased the IFNγ secretion of these TDLN-derived antigen-activated T cells (Figure 3l) and the expression of the degranulation marker, CD107a, on tumor-infiltrating T cells (Figure 3m). The increased secretion of IFNγ, along with elevated levels of activation and degranulation markers, suggests that H.rCCL2 not only enhances the migratory capacity of T cells but also promotes a more activated and responsive state in T cells, which could be crucial for effective anti-tumor immunity. We did not find any direct cytotoxic effects of H.rCCL2 on human bladder tumor cells (Supplementary Figure S8A), consistent with our murine data (Supplementary Figure S8B). This supports a mechanism of H.rCCL2-mediated anti-tumor effects through modulation of the immune response rather than direct tumor cell killing.

**Figure 4:**
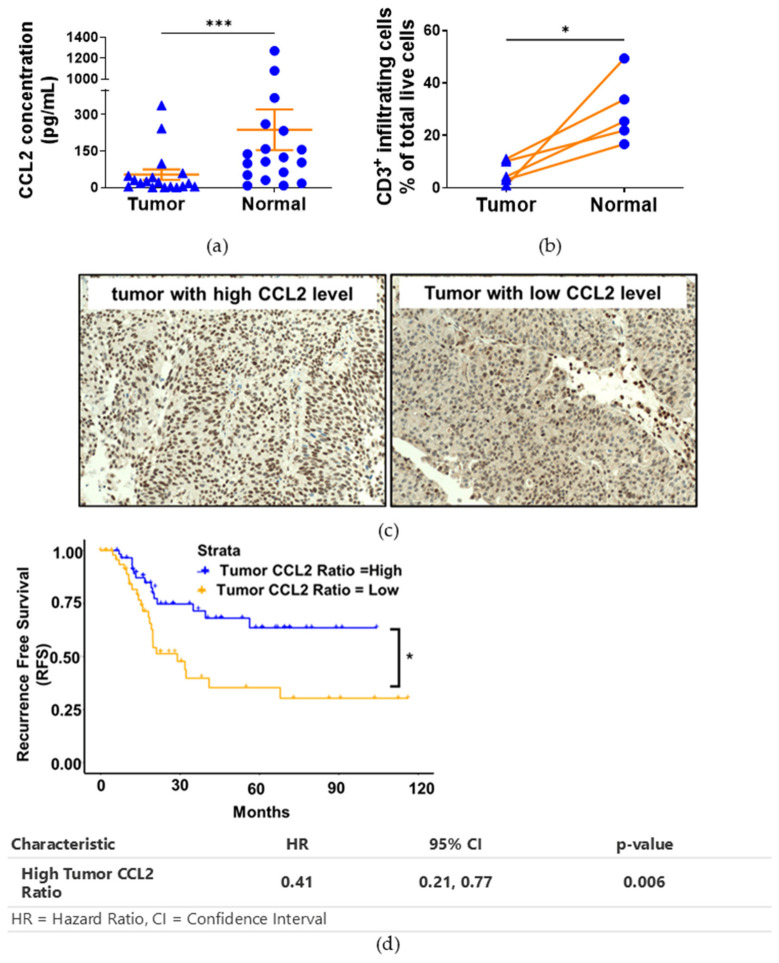

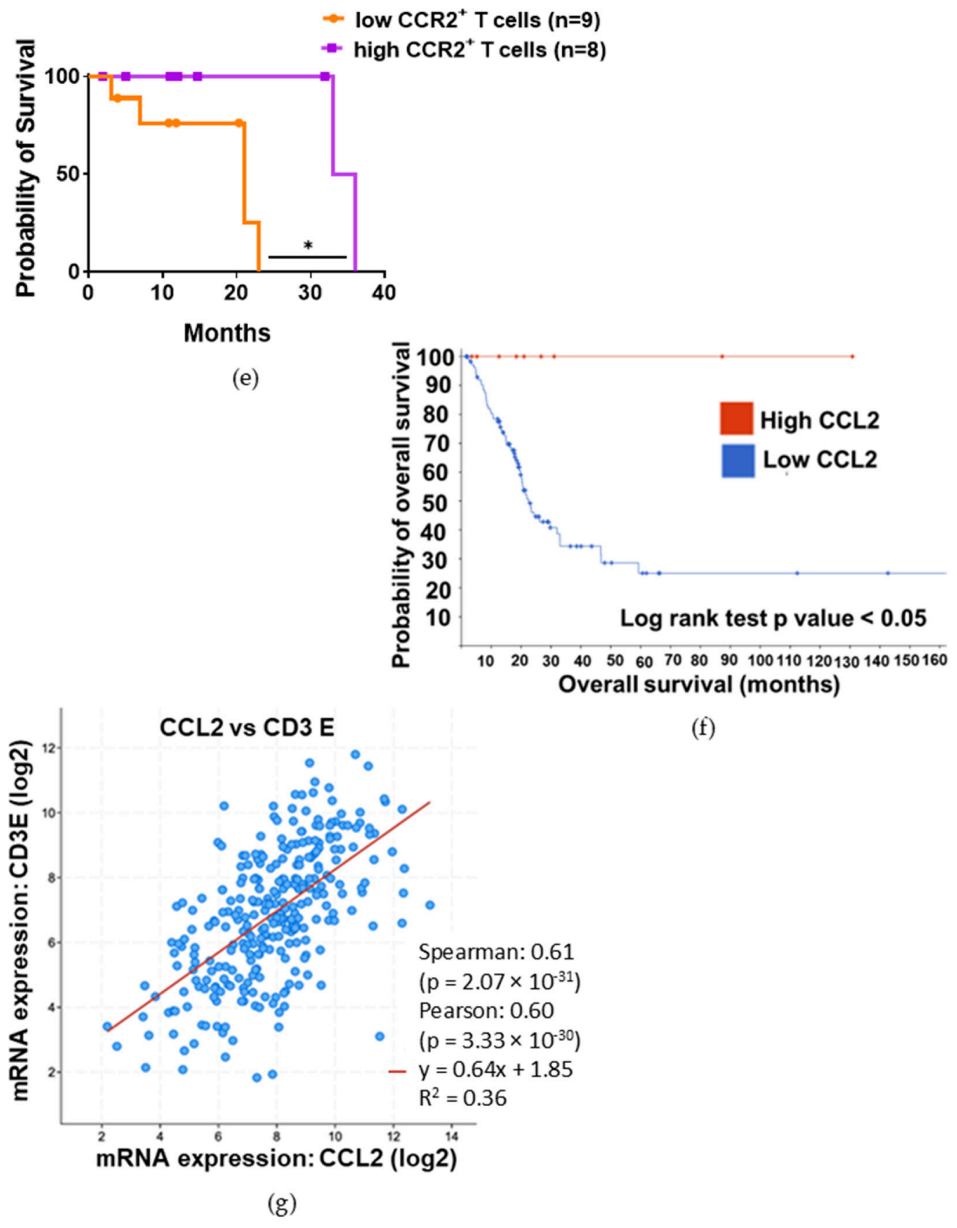
Significance of the CCL2/CCR2 anti-tumor axis in patients with BCa. (**a**) Bladder tumor tissue and adjacent normal tissue were processed into single-cell suspensions and cultured for 48 h to collect supernatant for human CCL2 detection by ELISA. Shown are the pooled results of 18 patients. *** *p* ≤ 0.001, unpaired *t*-test. (**b**) Bladder tumor tissue and adjacent normal tissue were collected from the same patient during the cystectomy procedure and processed into SCSs and analyzed by flow cytometry for infiltrating the CD3^+^ T-cell population out of total live cells. Shown are the pooled results of 5 patients (each line connecting two symbols represents matching tumor and adjacent normal samples from one patient). * *p* ≤ 0.05, paired *t*-test. (**c**,**d**) CCL2 staining was carried out in tumor tissues derived from patients with bladder cancer (*n* = 111). (**c**) Representative pictures of high and low CCL2 staining are shown (20× magnification). (**d**) CCL2 staining was evaluated based on the intensity (weak = 1, moderate = 2 and high = 3) of CCL2 staining and the density (0% = 0, 1–40% = 1, 41–75% = 2, >76% = 3). The final score of each sample was multiplied by the intensity and density. The tumor vs. CCL2 ratio was classified as high (>1) vs. low (≤1) based on whether the tumor CCL2 score was higher than the interphase CCL2 score. This was used as a categorical predictor in Cox proportional hazard regression, and a Kaplan–Meier curve was created to show the relative survival rates. The hazard ratio for high tumor CCL2 (>interphase CCL2) was statistically significant HR = 0.41 (95% CI 0.21, 0.77, *p* = 0.006). This indicates that a higher value of tumor CCL2 relative to the interphase CCL2 score had a lower risk of recurrence. (**e**) CCR2^+^ T cells detected in tumor tissues derived from patients with BCa (*n* = 17). Kaplan–Meier plot of recurrence-free survival (RFS) of patients according to intratumoral CCR2^+^ T-cell frequency. Associations with survival outcomes were examined by the population median and by splitting the population into two groups (high and low CCR2+ T cells). *p*-values represent the log-rank (Mantel–Cox) test, * *p* < 0.05. (**f**) Bladder tumors with CCL2 alterations are associated with higher overall patient survival in the TCGA BCa patient cohort. A Kaplan–Meier analysis was performed using cBioPortal for Cancer Genomics. The CCL2 high group includes patients with either CCL2 copy number changes or increased CCL2 mRNA expression. *p*-value = 0.0157, log-rank test. (**g**) Scatter plot and linear regression analysis of CD3E and CCL2 genes in bladder tumors of TCGA firehose legacy patient cohort. The Spearman correlation analysis of the expression of genes is based on RNA seq V2 RSEM data and obtained from cBioPortal for Cancer Genomics. The correlation is statistically significant (*p* < 0.05).

To further investigate the human relevance of the CCL2/CCR2 axis in BCa, data from TCGA and a local patient cohort was analyzed. Supernatants from tumor tissues of local patients with BCa had lower CCL2 levels and a lower number of T cells compared to supernatants taken from adjacent normal tissue (Figure 4a,b). Similarly, we observed a significant reduction in CCL2 expression when comparing the TCGA bladder tumor dataset with the Genotype–Tissue Expression (GTEx) normal bladder dataset (Supplementary Figure S9A). CCL2 levels were also decreased in MB49 mouse bladder tumors relative to normal mouse bladder tissue (Supplementary Figure S9B). Exposure to the BBN carcinogen for 3 months resulted in a reduction in CCL2 levels compared to a shorter 1-month exposure (Supplementary Figure S9C). These results suggest that cancer development modulates CCL2 levels in the bladder, with higher levels present in normal bladder urothelium compared to BCa, aligning with the anti-tumor effects of CCL2 in the bladder.

To assess the clinical significance of human bladder tumor CCL2, CCL2 staining was performed on pre-treatment bladder tumor tissue from a cohort of patients with non-muscle-invasive BCa who were treated with intravesical BCG following tumor resection. Patient characteristics are shown in Table 1. Compared to patients with lower tumor CCL2, patients with higher tumor CCL2 levels experienced improved recurrence-free survival (RFS) (Figure 4c,d). This association remained significant after adjusting for patient age and sex and tumor stage (Table 2). In a separate BCa cohort (Table 3), from which tumor-infiltrating lymphocytes were available for analysis, the presence of bladder-infiltrating CCR2^+^ T cells was associated with improved patient survival (Figure 4e). These findings were validated in the TCGA cohort, where patients with high tumor CCL2 had significantly improved survival compared to patients with low tumor CCL2 (Figure 4f). Further, in the TCGA cohort, CCL2 expression positively correlated with T cells (marked by CD3 expression), indicating a link between CCL2 and T-cell infiltration (Figure 4g). In conclusion, these data indicate that CCL2 is lower in BCa tissues compared to normal bladder tissues, and higher CCL2 levels in tumor tissues are associated with improved survival outcomes.

## 4. Discussion

Our findings reveal a context-dependent anti-tumor role for CCL2 in BCa. While CCL2 is traditionally associated with tumor-promoting roles through recruitment of pro-tumorigenic myeloid cells [14,15,16,17,18], we demonstrate that, in BCa, CCL2 plays a T-cell-dependent anti-tumoral role. The loss of CCL2 or CCR2 resulted in accelerated tumor growth and increased tumor incidence in multiple preclinical BCa models, emphasizing the critical importance of this axis in tumor suppression. Clinically, high tumor CCL2 levels were associated with improved survival in patients with BCa, validating the protective role of CCL2 in BCa. While previous studies focused on blockade of the CCL2 axis as a therapeutic strategy [53,54], we demonstrated that intravesical instillation of rCCL2 restored CCR2^+^ T-cell recruitment to the bladder tumor environment and enhanced their effector function, leading to tumor size reduction and improved survival in both syngeneic and humanized models of BCa. These findings support a nuanced role of CCL2 in cancer biology with a promising therapeutic avenue for BCa.

Although the role of CCL2 in the bladder TME has not been extensively studied, some investigations have explored its relevance in BCa. Urinary levels of human CCL2 were correlated with bladder tumor stage, grade, and metastasis [55], while blocking the CCL2/CCR2 axis and anti-PD-1 therapy enhanced immune checkpoint responses in subcutaneous murine BCa [53]. This contrasts with our findings and could be due to differences in model systems. Compared to the subcutaneous BCa model, the orthotopic model used here may provide a more relevant model for understanding human bladder-specific immune contexts. Another study identified the long noncoding RNA LNMAT1 as a driver of lymphatic metastasis via epigenetic activation of CCL2, recruiting tumor-associated macrophages to promote metastasis [56], showing that the role of CCL2 may also vary with tumor stage and progression such as between primary and metastatic settings. Also, the precise cellular source of CCL2 (e.g., urothelial, myeloid, stromal), disease stage, and nodal involvement can lead to differential prognostic outcomes. This has been demonstrated by a recent study showing that tumor cell-derived CCL2 led to poor clinical outcomes, whereas immune cell-derived CCL2 was linked with improved prognosis, contingent on nodal status [31]. Collectively, these findings highlight the stage and context-dependent functions of CCL2 in BCa.

Our study also found that αCCL2 action was tissue-specific; αCCL2 enhanced bladder tumor growth, in contrast to its therapeutic effect on mammary tumor growth, underscoring the microenvironment-specific role of CCL2 in cancer. Several recent studies in other cancers support a dual role for CCL2 in cancer development. Li et al. showed that CCL2 has a biphasic role in breast cancer and blocking CCL2 reduces immunosurveillance during cancer initiation [27]. CCL2 was also found to be a favorable prognostic factor in pancreatic cancer [57], sarcoma, and non-small-cell lung cancer patients [58,59]. These contrasting observations, deviating from the established pro-cancer role of CCL2, highlight the complexity of targeting this pathway uniformly across tumor types and emphasize the need for a thorough investigation of the CCL2/CCR2 pathway in specific tumor microenvironments before pursuing CCL2 blockade as a universal anti-cancer strategy.

Lastly, we also investigated a new therapeutic strategy, introducing rCCL2 as a recombinant chemokine therapeutic agent—the first chemokine-based intravesical agent in BCa—rather than as an antagonist. In this study, intravesical administration of rCCL2 demonstrated promising anti-tumor efficacy, and combination therapy with gemcitabine and rCCL2 was more effective than either agent alone. Our results offer a distinct yet synergistic approach compared to recent FDA-approved therapies such as Anktiva, an IL-15 receptor agonist enhancing systemic NK- and T-cell responses [60], and Adstiladrin, an intravesical gene therapy promoting local immune activation [61]. By addressing poor immune infiltration and exhaustion associated with BCG-unresponsive disease [62] and immune checkpoint inhibitor failure [63], the promising anti-tumor effects position rCCL2 as a potential standalone therapy or adjunct to existing treatments, offering a new strategy to improve BCa outcomes.

There were limitations to this investigation. The dual role of CCL2 in BCa, similar to its biphasic function observed in other cancers like breast, remains incompletely understood. Future studies should focus on clarifying these complexities, particularly by distinguishing the differential roles of CCL2 during the early and late stages of BCa progression and further investigating the contribution of myeloid cells and other CCR2-responsive populations within the bladder tumor microenvironment. Secondly, while we utilized diverse BCa murine models, they did not fully capture the heterogeneity and complexity of human BCa. Additionally, our humanized models, though valuable for testing the H.rCCL2-mediated immune cell infiltration, represent artificial systems that may not entirely reflect human TME. Similarly, while our BBN model in immunocompetent mice is meant to mimic tobacco-induced carcinogenesis in humans, it does not fully recapitulate all of the complex carcinogens found in tobacco. However, we tested rCCL2 in other immunocompetent murine BCa models and clinical samples to address these limitations. Another constraint in the field is the incomplete characterization of CCL2’s cellular sources within the bladder TME, which could influence interpretations of how various cell types contribute to this signaling axis. Future research will explore these cellular sources utilizing techniques better suited for cellular analyses such as spatial profiling, multiplex IHC, RNA in situ hybridization, or single-cell sequencing to better understand their roles in tumor suppression within the bladder TME. Finally, while we identified CCL2/CCR2-mediated T-cell recruitment and activation as a key mechanism, its interactions with resident immune cells, such as NK cells and stromal cells, remain under investigation. This, as well as the possibility of toxicity including systemic inflammation, must be further investigated, with proper pharmacokinetic and toxicity studies, to fully ascertain the therapeutic potential of rCCL2 in BCa.

## 5. Conclusions

In conclusion, this study identifies a context-dependent, tumor-suppressive role for the CCL2/CCR2 axis in bladder cancer, in which CCL2 promotes CCR2^+^ T-cell recruitment, activation, and anti-tumor function. These findings challenge the view of CCL2 as uniformly tumor-promoting and highlight the importance of evaluating chemokine biology within specific tumor microenvironments. By demonstrating that intravesical rCCL2 enhances T-cell infiltration and improves tumor control in preclinical and humanized bladder cancer models, this work demonstrates the efficacy of rCCL2-based therapy in conjunction with chemotherapy as a novel immune-enhancing strategy for treatment-resistant bladder cancer. Future studies focusing on the cellular source of CCL2, as well as the safety of intravesical administration of rCCL2, are crucial in establishing the clinical utility of rCCL2.

## Figures and Tables

**Table 1:** Patient characteristics of BCa patient cohort CCL2 staining scores *n* = 111.

**Mean** **(Range) Age**		73 (31–92) years
**Sex**	Female	18 (16.2%)
	Male	93 (83.8%)
Tumor Stage *	Ta	34 (30.6%)
	T1	60 (54.1%)
	CIS	2 (1.8%)
	Ta/CIS	4 (3.6%)
	T1/CIS	11 (9.9%)
**Histological Subtype**	None	104 (93.7%)
	Squamous	4 (3.6%)
	Micropapillary	1 (0.9%)
	Glandular	1 (0.9%)
	Other	1 (0.9%)

* All tumor samples were high grade at the time of TURBT.

**Table 2:** Multivariate analysis of CCL2 staining.

Characteristic	HR *	95% CI *	*p*-Value
Age	1.00	0.96, 1.04	>0.9
Male	1.05	0.40, 2.73	>0.9
T-Stage	0.88	0.66, 1.19	0.4
Tumor CCL2 Ratio			
Low	-	-	
High	0.39	0.19, 0.79	0.009

* HR = hazard ratio; CI = confidence interval.

**Table 3:** Patient characteristics listed for the patient cohort (*n* = 17) used for CCR2^+^ T-cell analysis by flow cytometry (Figure 4e).

**Mean** **(Range) Age**		69.6 (47–86) years
**Sex**	Female	6 (35.3%)
	Male	11 (64.7%)
**Tumor Stage**	T2	2 (11.8%)
	T3	6 (35.3%)
	T4	9 (52.9%)
**Histological Subtype**	Urothelial	12 (70.6%)
	Squamous	3 (17.6%)
	Urothelial/Squamous	1 (5.9%)
	Sarcomatoid	1 (5.9%)

## Data Availability

Data is available upon request.

## Supplementary Materials

The following supporting information can be downloaded at: https://www.mdpi.com/article/10.3390/cancers18142267/s1, Figure S1: Anti-CCL2 (αCCL2) is therapeutic in mammary tumors. WT B6 mice were injected with 0.5 × 10^6^ AT-3 (A) and MB49 (B) cells into the 4th mammary fat pad and treated intraperitoneally with αCCL2 10 mg/kg, starting from day 1 after tumor challenge, twice a week. Tumor growth was monitored over 4 weeks. ns; not significant, * *p* ≤ 0.05, two-way ANOVA; Figure S2: T-cell depletion abrogates CCL2-mediated anti-tumor effects in BCa. (A) WT and CCL2^−/−^ mice were challenged orthotopically with 8 × 10^4^ MB49 cells and sacrificed after ~3 weeks for bladder tumor processing. MB49 tumor-challenged WT and CCL2^−/−^ mice were injected intraperitoneally with 250 μg anti-CD4 or anti-CD8 antibodies twice weekly starting one day before the tumor challenge. Mice were sacrificed at ~3 weeks for determination of bladder tumor weights. Mean ± SEM; ns; not significant, unpaired *t*-test; Figure S3: Increased bladder tumor weights are observed upon CCR2 knockout in BBN-mediated model of bladder carcinogenesis. (A–C) WT C57BL/6 (WT) and CCR2 KO (CCR2^−/−^) mice were given BBN in drinking water for 4.5 months to induce bladder carcinogenesis. Mice were sacrificed and bladders were weighed (A) and processed for histopathologic examination of bladders. Mean ± SEM; ** *p* < 0.01, Mann–Whitney. (B) Carcinogenesis was measured by the pathological incidences. Normal—no cancer; pT1/pT2—cancer. (C) Urothelium was classified as normal urothelium, pT1 tumor (tumor has spread to the lamina propria), or pT2 tumor (tumor has invaded the muscle); Figure S4: Frequency of CCR2^+^ and CCR2^−^ T cells in spleen and lymph node in mixed bone marrow chimera experiment. CD45.1 CCR2^+^/CD45.2 CCR2^−/−^ bone marrow chimera mice were challenged orthotopically as described in the Materials and Methods and sacrificed at ~3 weeks. Tumor-draining lymph nodes (A) and spleens (B) were harvested and processed for immune analysis and T cells were detected by flow cytometry. Mean ± SEM; **** *p* ≤ 0.0001, unpaired *t*-test; Figure S5: CCR2^+^ T were more functional compared with CCR2^−^ T cells in bladder tumors. CD45.1 CCR2^+^//CD45.2 CCR2^−/−^ bone marrow chimera mice were challenged orthotopically as described in the Materials and Methods and sacrificed at ~3 weeks. Bladders were harvested and processed for immune analysis and CD25^+^ T cells (A) and tetramer^+^ T cells (B) were detected by flow cytometry. Mean ± SEM; * *p* ≤ 0.05, ** *p* < 0.01, *** *p* < 0.001, Mann–Whitney. The level of expression of IFNγ (C), perforin (D), and granzyme B (E) on bladder tumor-infiltrating T cells was calculated by MFI based on flow cytometry data. Mean ± SEM; *** *p* < 0.001, unpaired *t*-test; Figure S6: Recombinant CCL2 decreases SQ MB49 tumor growth. WT mice were challenged subcutaneously with 2 × 10^5^ MB49 cells. Recombinant CCL2 (rCCL2) (20 μg/kg) or PBS was intratumorally injected on day 7, then every 5 days. Tumor growth was monitored over 3 weeks. * *p* ≤ 0.05, two-way ANOVA; Figure S7: CCL2 increases the gene expression in T cells associated with T-cell activation, proliferation, cytokine responses, and proinflammatory response. As described in the Materials and Methods, CD4^+^ and CD8^+^ T cells were sorted from (A,B) PBMCs or (C,D) TDLNs pooled from at least 3 patients with BCa, then co-cultured with 800 ng/mL of human recombinant CCL2 or with medium only as a control for 24 h prior to RNA extraction and gene expression analysis. Shown is the volcano plot indicating changes in the gene expression of (A,C) cytokines or chemokines and (B,D) cytokine receptors or cell surface markers related to activated T cells or proinflammatory response. (E,F) CD4^+^ and CD8^+^ T cells were sorted from PBMCs or TDLNs pooled from at least 3 patients with BC, then co-cultured with 800 ng/mL of human recombinant CCL2 or with medium only as a control for 24 h prior to RNA extraction and gene expression analysis. Shown are the heat maps indicating differential expression of genes associated with (E) proliferation or (F) cytokine production in effector T cells; Figure S8: rCCL2 shows no direct cytotoxicity effect on tumor cells in vitro. (A) T24 cells were cultured with or without the presence of human recombinant CCL2 (H.rCCL2) at 800 ng/mL for 24 h. The viability of cells was measured by flow cytometry analysis after FVD staining (unpaired *t*-test). (B) MB49 cells were cultured with or without the presence of mouse recombinant CCL2 (rCCL2) at 800 ng/mL for 24 h in triplicate or quadruplicate. The viability of cells was measured by flow cytometry analysis after FVD staining as described in the Materials and Methods. Shown are the representative results from two independent experiments. Mean ± SEM; ns; not significant, unpaired *t*-test; Figure S9: CCL2 is downregulated in mouse and human BCa. (A) Gene expression profiling interactive analysis (GEPIA) with TCGA BLCA and GTEx datasets was used to calculate differences in CCL2 expression in bladder tumor samples (*n* = 404) compared with normal bladder samples (*n* = 28). The red box represents the cancer tissue group, the gray box represents the normal tissue group, and the asterisk represents *p* < 0.01. The dots represented expression in each sample. (B) WT mice were challenged orthotopically with 8 × 10^4^ MB49 cells and sacrificed after ~3 weeks for bladder tumor processing. In total, 0.5 × 10^6^ bladder cells were plated in 50 μL in a 96-well plate. The supernatant was collected after 24 h and CCL2 level was measured by a mouse chemokine array. Relative CCL2 protein expression was normalized with HSP60 control protein expression. (C) WT C57BL/6 mice were given BBN in drinking water for 1 month (control) and 3 months (tumor induction by chronic exposure). Mice were sacrificed, bladders were processed and 0.5 × 10^6^ bladder cells were plated in 50 μL in a 96-well plate. The supernatant was collected after 24 h and the CCL2 level was measured by a mouse chemokine array. Relative CCL2 protein expression was normalized with HSP60 control protein expression. Mean ± SEM; * *p* < 0.05, ** *p* < 0.01, unpaired *t*-test.
