# Supplementary material for: Unveiling the Paradoxical Tumor-Suppressive Role of CCL2/CCR2 in Bladder Cancer: A Novel Immunotherapeutic Strategy
Source: Cancers (Basel). 2026 Jul 15;18(14):2267. doi: 10.3390/cancers18142267 (PMC13406517; doi:10.3390/cancers18142267)
Supplement: Supplementary file 1 [file cancers-18-02267-s001.zip › cancers-4423057-supplementary.pdf]

Supplementary Materials: Unveiling the Paradoxical Tumor-Suppressive Role of CCL2/CCR2 in Bladder Cancer: A Novel Immunotherapeutic Strategy

Neelam Mukherjee, Niannian Ji, Zaineb Hassouneh, Jaime Furman, Olivia Fisher, Jonathan Gelfond, Onika D. V. Noel, Gisele Morales, Xi Tan, Chun-Liang Chen, Solomon L. Woldu, Yair Lotan and Robert S. Svatek

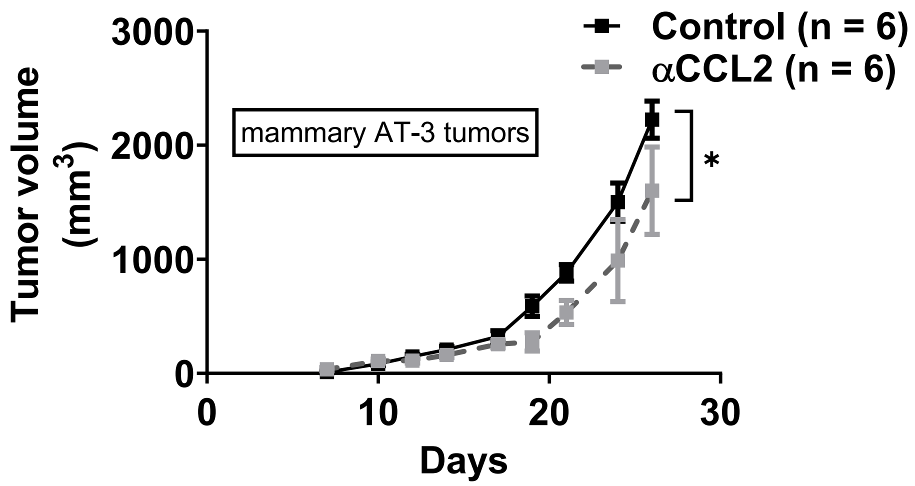

(**A**)

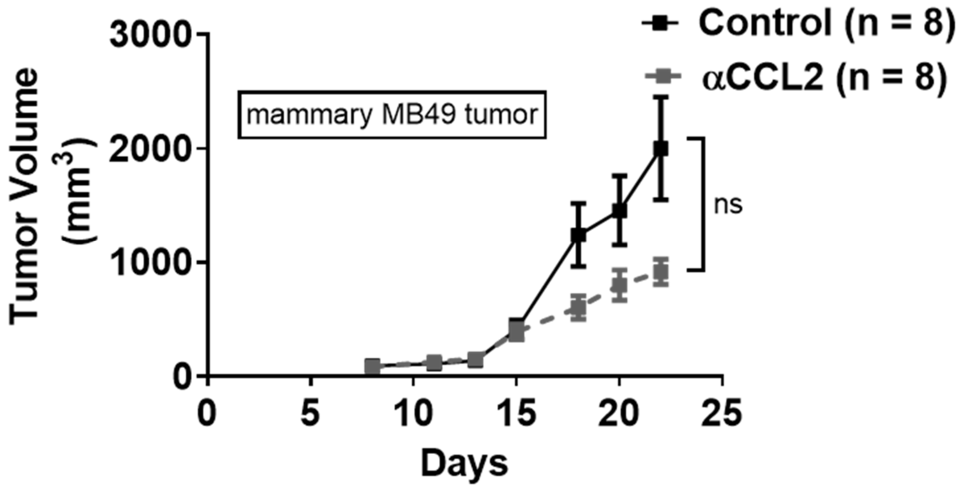

(**B**)

Supplementary Figure S1. Anti-CCL2 (αCCL2) is therapeutic in mammary tumors. WT B6 mice were injected with 0.5×10^6 AT-3 (**A**) and MB49 (**B**) cells into the 4th mammary fat pad and treated intraperitoneally with αCCL2 10 mg/kg, starting from day 1 after tumor challenge, twice a week. Tumor growth was monitored over 4 weeks. ns; not significant, * $p \leq 0.05$, two-way ANOVA.

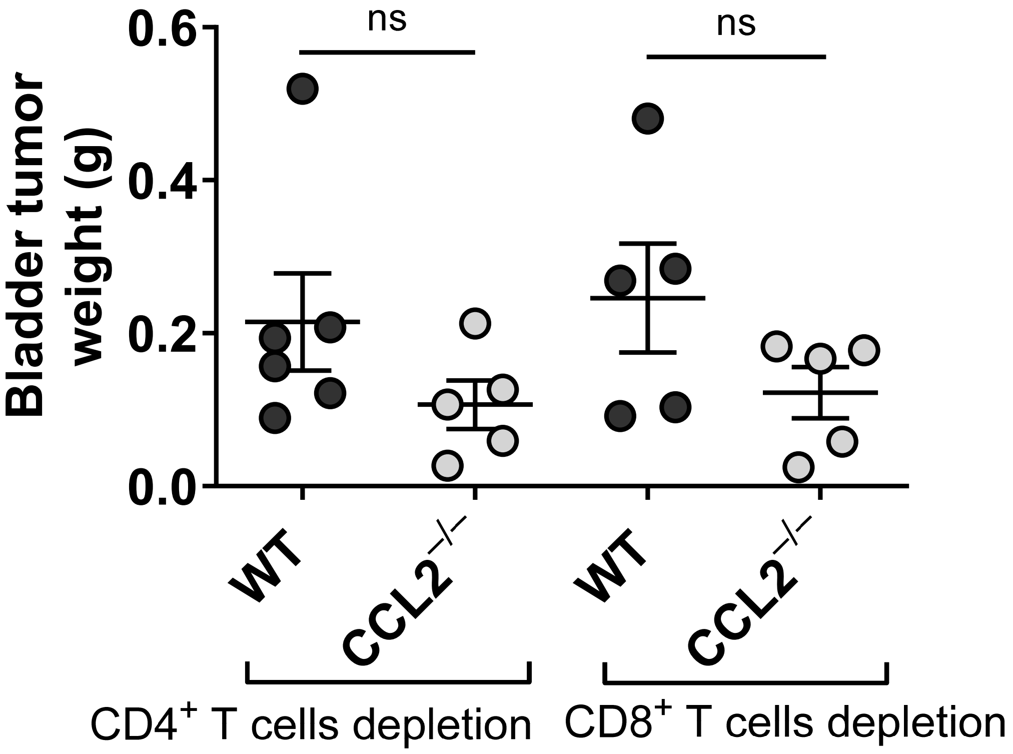

Supplementary Figure S2. T-cell depletion abrogates CCL2-mediated anti-tumor effects in BCa. (**A**) WT and CCL2$^{-/-}$ mice were challenged orthotopically with 8 × 10^{4} MB49 cells and sacrificed after ~3 weeks for bladder tumor processing. MB49 tumor-challenged WT and CCL2$^{-/-}$ mice were injected intraperitoneally with 250 μg anti-CD4 or anti-CD8 antibodies twice weekly starting one day before the tumor challenge. Mice were sacrificed at ~3 weeks for determination of bladder tumor weights. Mean ± SEM; ns; not significant, unpaired *t*-test.

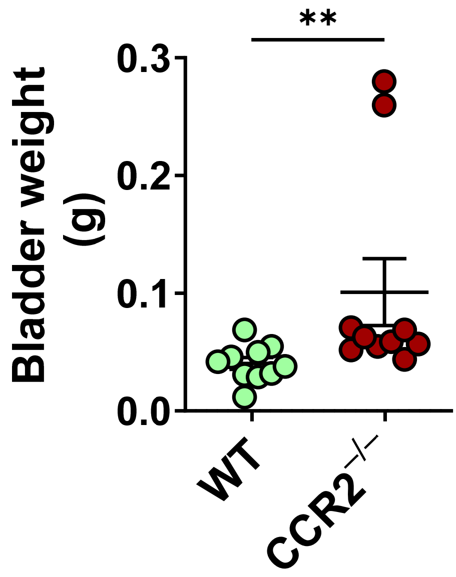

(A)

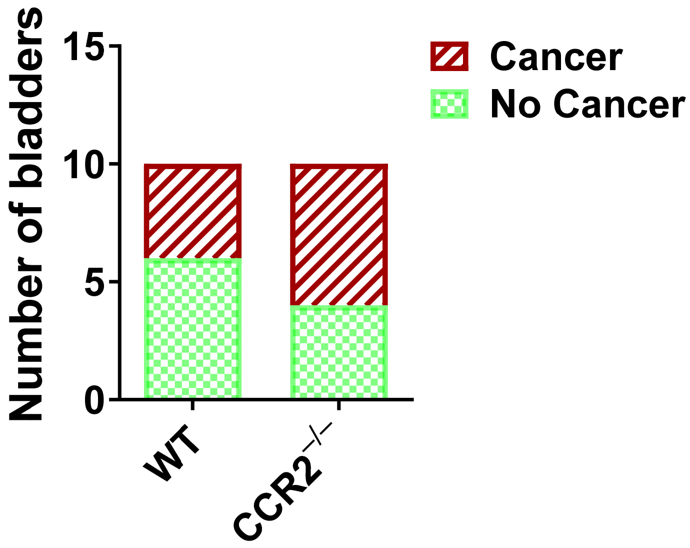

(B)

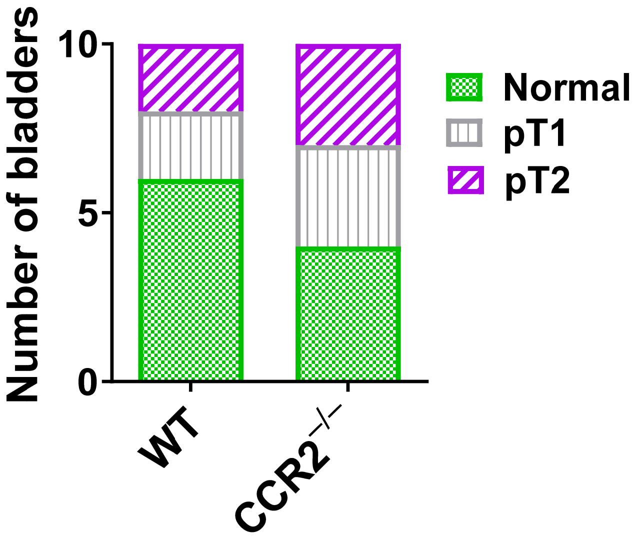

(C)

Supplementary Figure S3. Increased bladder tumor weights are observed upon CCR2 knockout in BBN-mediated model of bladder carcinogenesis. (**A**–**C**) WT C57BL/6 (WT) and CCR2 KO (CCR2$^{-/-}$) mice were given BBN in drinking water for 4.5 months to induce bladder carcinogenesis. Mice were sacrificed and bladders were weighed (**A**) and processed for histopathologic examination of bladders. Mean ± SEM; ** $p < 0.01$, Mann–Whitney. (**B**) Carcinogenesis was measured by the pathological incidences. Normal—no cancer; pT1/pT2—cancer. (**C**) Urothelium was classified as normal urothelium, pT1 tumor (tumor has spread to the lamina propria), or pT2 tumor (tumor has invaded the muscle).

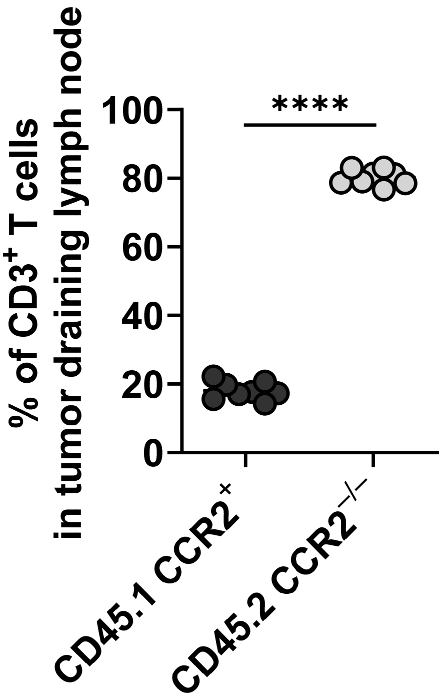

(**A**)

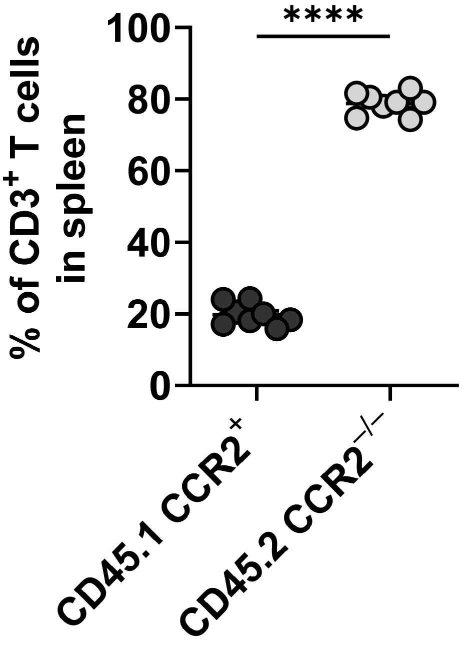

(**B**)

Supplementary Figure S4. **Frequency of CCR2^{+} and CCR2^{-} T cells in spleen and lymph node in mixed bone marrow chimera experiment.** CD45.1 CCR2^{+}/CD45.2 CCR2$^{-/-}$ bone marrow chimera mice were challenged orthotopically as described in the Materials and Methods and sacrificed at ~3 weeks. Tumor-draining lymph nodes (**A**) and spleens (**B**) were harvested and processed for immune analysis and T cells were detected by flow cytometry. Mean ± SEM; **** $p \leq 0.0001$, unpaired *t*-test.

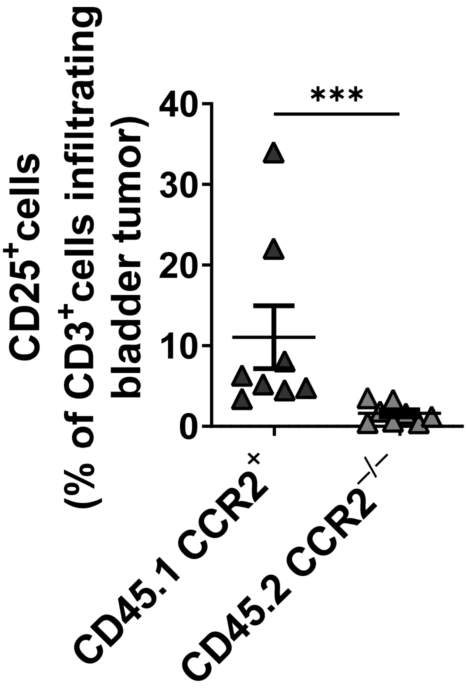
CD25+cells
(% of CD3+cells infiltrating
bladder tumor)
40
30
20
10
0
***
CD45.1 CCR2+
CD45.2 CCR2−/−

(A)

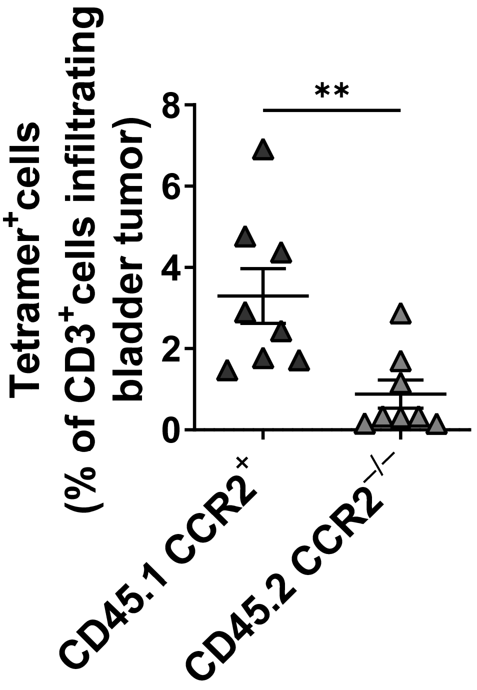
Tetramer+cells
(% of CD3+cells infiltrating
bladder tumor)
8
6
4
2
0
**
CD45.1 CCR2+
CD45.2 CCR2−/−

(B)

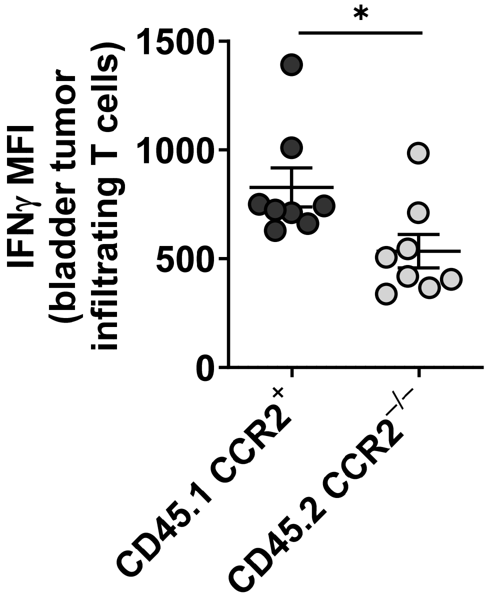
IFNγ MFI
(bladder tumor
infiltrating T cells)
1500
1000
500
0
*
CD45.1 CCR2+
CD45.2 CCR2−/−

(C)

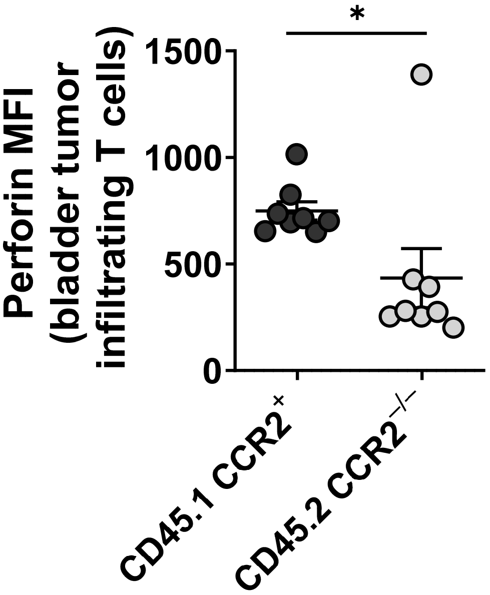
Perforin MFI
(bladder tumor
infiltrating T cells)
1500
1000
500
0
*
CD45.1 CCR2+
CD45.2 CCR2−/−

(D)

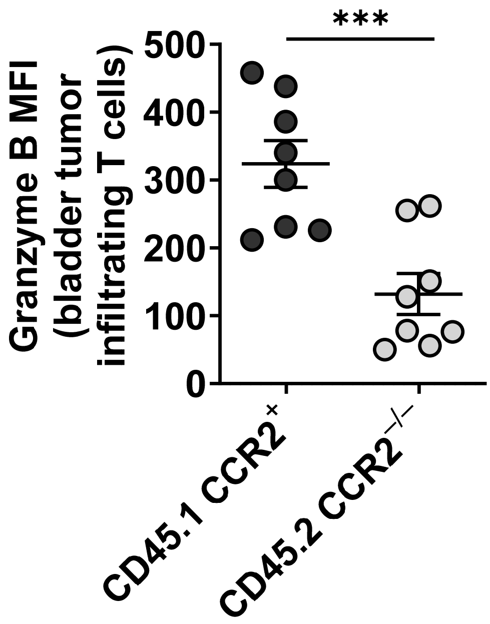

(E)

Supplementary Figure S5. **CCR2⁺ T were more functional compared with CCR2⁻ T cells in bladder tumors.** CD45.1 $CCR2^{+}$//CD45.2 $CCR2^{-/-}$ bone marrow chimera mice were challenged orthotopically as described in the Materials and Methods and sacrificed at ~3 weeks. Bladders were harvested and processed for immune analysis and $CD25^{+}$ T cells (**A**) and $tetramer^{+}$ T cells (**B**) were detected by flow cytometry. Mean ± SEM; * $p \leq 0.05$, ** $p < 0.01$, *** $p < 0.001$, Mann–Whitney. The level of expression of IFNγ (**C**), perforin (**D**), and granzyme B (**E**) on bladder tumor-infiltrating T cells was calculated by MFI based on flow cytometry data. Mean ± SEM; *** $p < 0.001$, unpaired *t*-test.

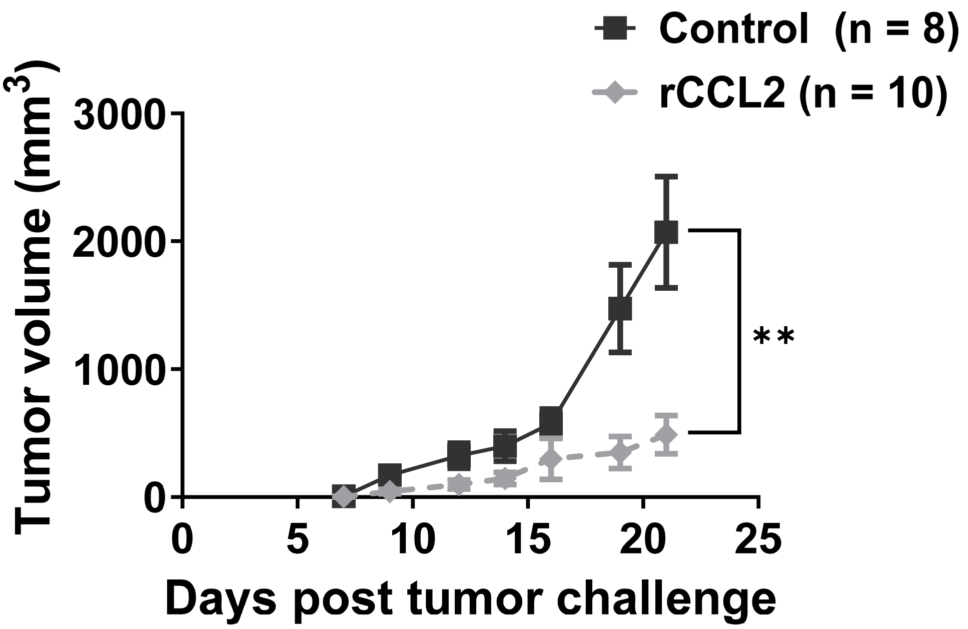

Supplementary Figure S6. Recombinant CCL2 decreases SQ MB49 tumor growth. WT mice were challenged subcutaneously with 2 × 10^5 MB49 cells. Recombinant CCL2 (rCCL2) (20 μg/kg) or PBS was intratumorally injected on day 7, then every 5 days. Tumor growth was monitored over 3 weeks. * $p \leq 0.05$, two-way ANOVA.

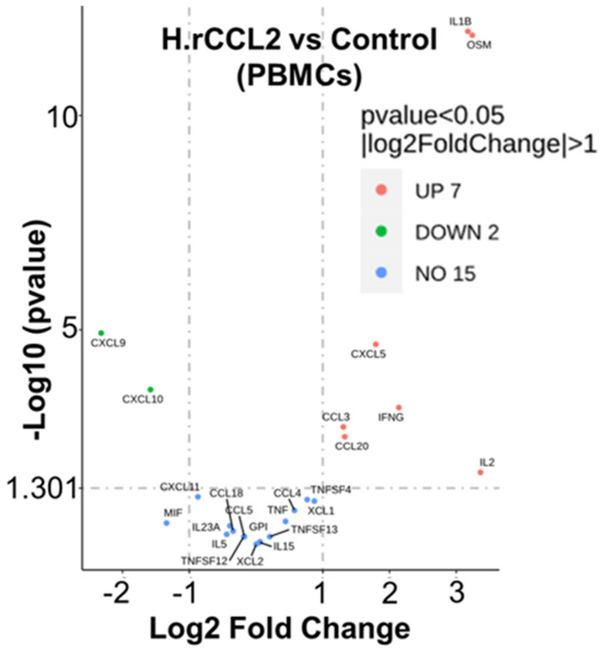
H.rCCL2 vs Control
(PBMCs)
pvalue<0.05
|log2FoldChange|>1
UP 7
DOWN 2
NO 15
-Log10 (pvalue)
Log2 Fold Change

(A)

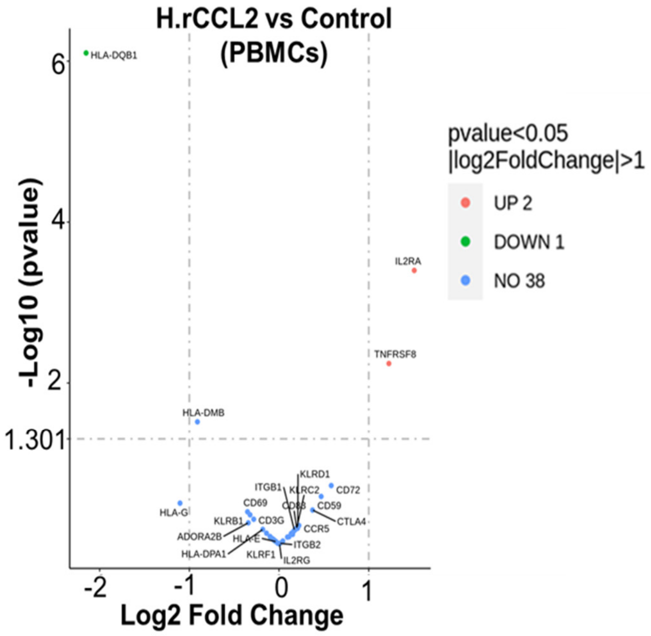
H.rCCL2 vs Control
(PBMCs)
pvalue<0.05
|log2FoldChange|>1
UP 2
DOWN 1
NO 38
-Log10 (pvalue)
Log2 Fold Change

(B)

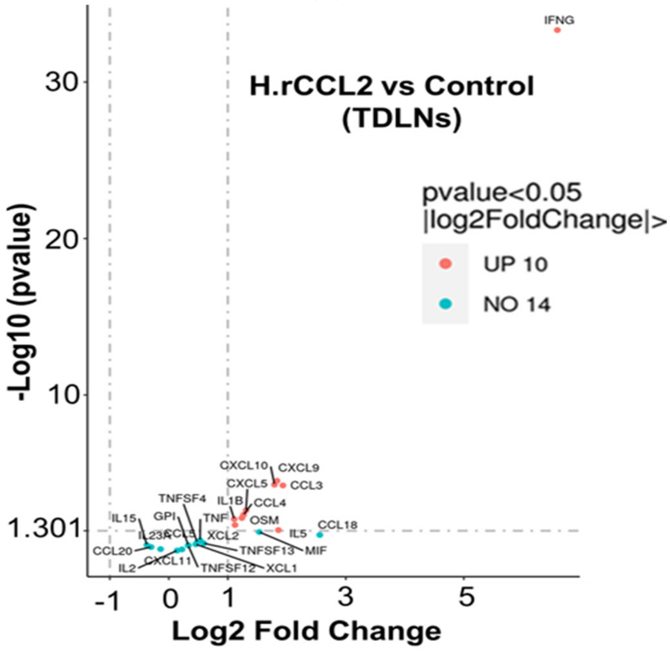
H.rCCL2 vs Control
(TDLNs)
pvalue<0.05
|log2FoldChange|>
UP 10
NO 14
-Log10 (pvalue)
Log2 Fold Change

(C)

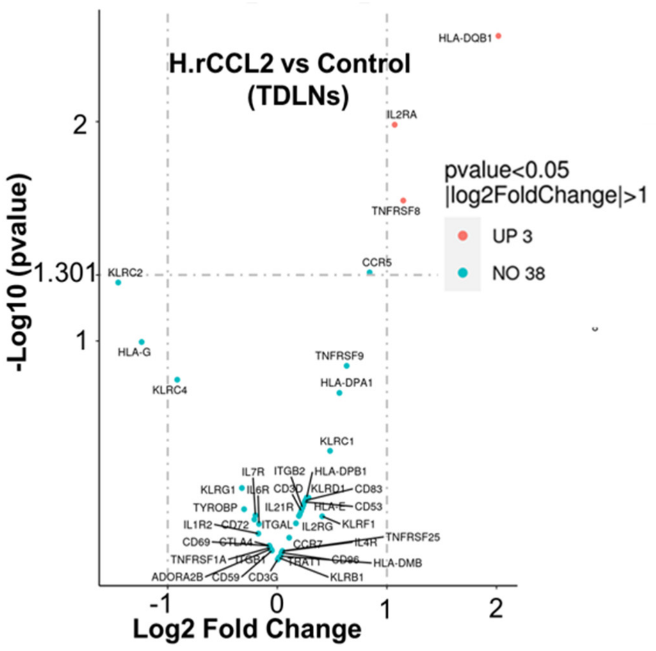
H.rCCL2 vs Control
(TDLNs)
pvalue<0.05
|log2FoldChange|>1
UP 3
NO 38
-Log10 (pvalue)
Log2 Fold Change

(D)

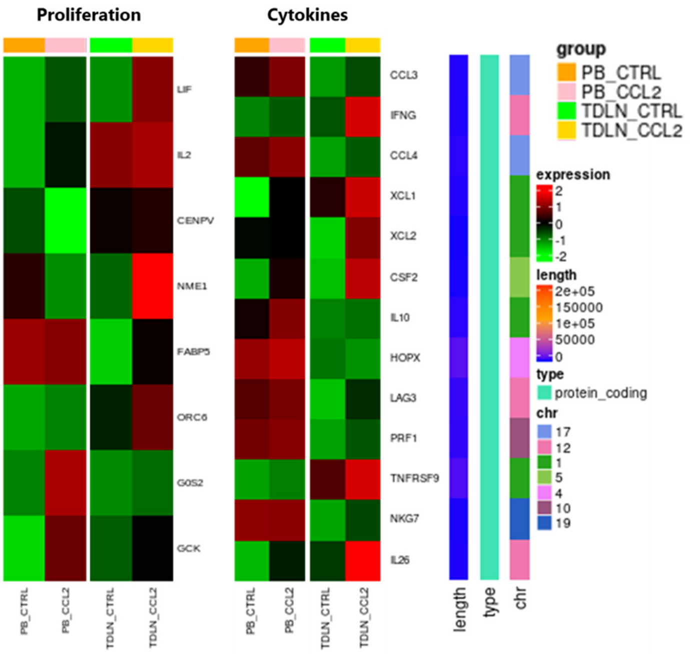

(E) (F)

Supplementary Figure S7. CCL2 increases the gene expression in T cells associated with T-cell activation, proliferation, cytokine responses, and proinflammatory response. As described in the Materials and Methods, CD4$^+$ and CD8$^+$ T cells were sorted from (**A**,**B**) PBMCs or (**C**,**D**) TDLNs pooled from at least 3 patients with BCa, then co-cultured with 800 ng/mL of human recombinant CCL2 or with medium only as a control for 24 h prior to RNA extraction and gene expression analysis. Shown is the volcano plot indicating changes in the gene expression of (**A**,**C**) cytokines or chemokines and (**B**,**D**) cytokine receptors or cell surface markers related to activated T cells or proinflammatory response. (**E**,**F**) CD4$^+$ and CD8$^+$ T cells were sorted from PBMCs or TDLNs pooled from at least 3 patients with BC, then co-cultured with 800 ng/mL of human recombinant CCL2 or with medium only as a control for 24 h prior to RNA extraction and gene expression analysis. Shown are the heat maps indicating differential expression of genes associated with (**E**) proliferation or (**F**) cytokine production in effector T cells.

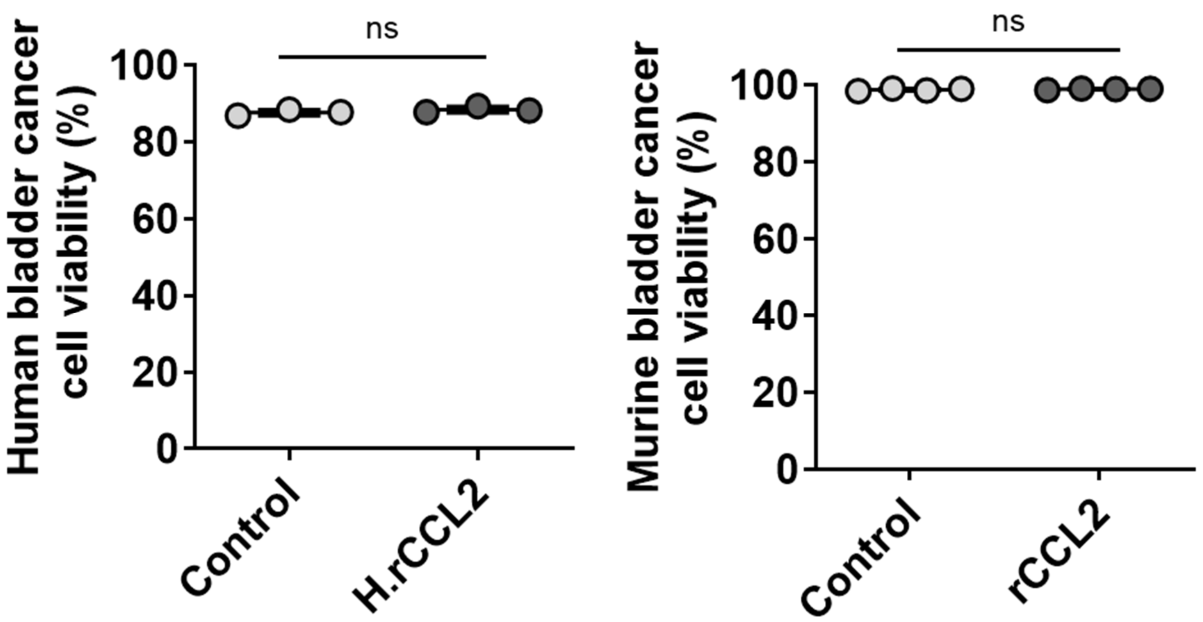

(A) **(B)**

Supplementary Figure S8. rCCL2 shows no direct cytotoxicity effect on tumor cells in vitro. (**A**) T24 cells were cultured with or without the presence of human recombinant CCL2 (H.rCCL2) at 800 ng/mL for 24 h. The viability of cells was measured by flow cytometry analysis after FVD staining (unpaired *t*-test). (**B**) MB49 cells were cultured with or without the presence of mouse recombinant CCL2 (rCCL2) at 800 ng/mL for 24 h in triplicate or quadruplicate. The viability of cells was measured by flow cytometry analysis after FVD staining as described in the Materials and Methods. Shown are the representative results from two independent experiments. Mean ± SEM; ns; not significant, unpaired *t*-test.

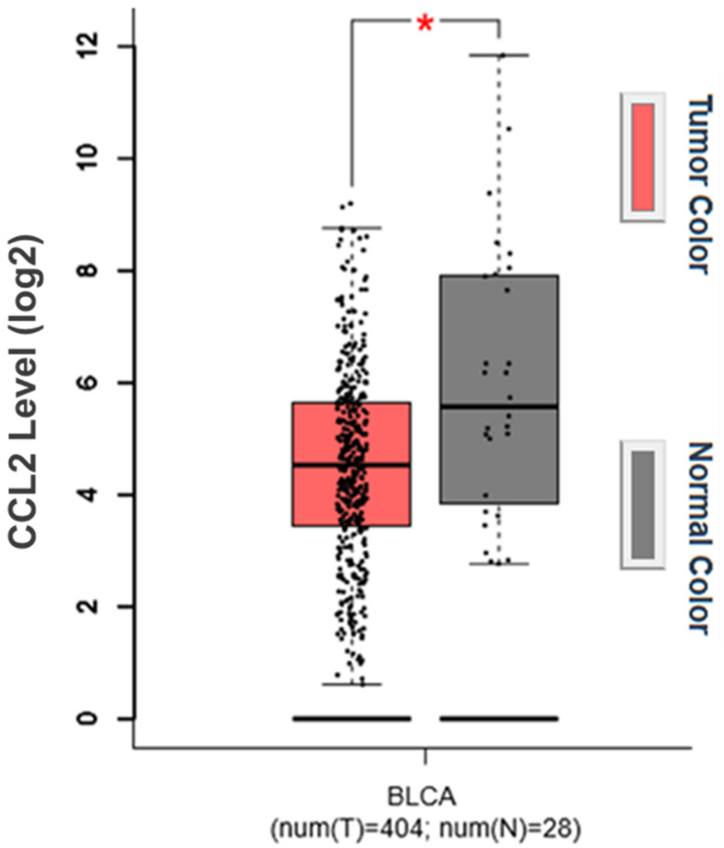

(**A**)

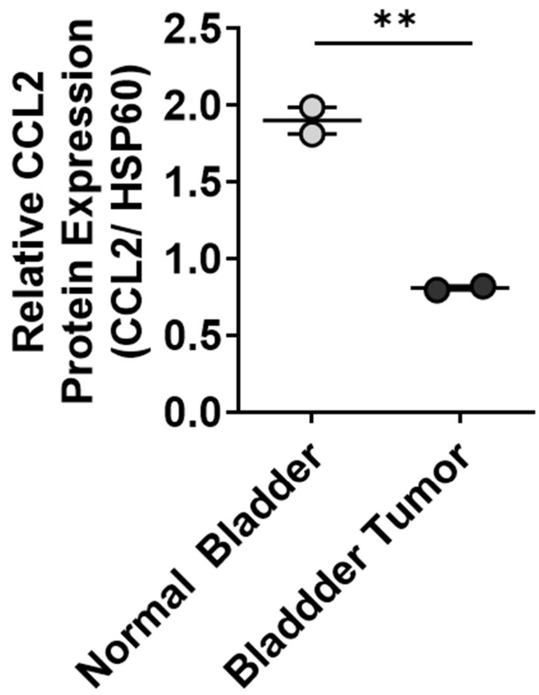

(**B**)

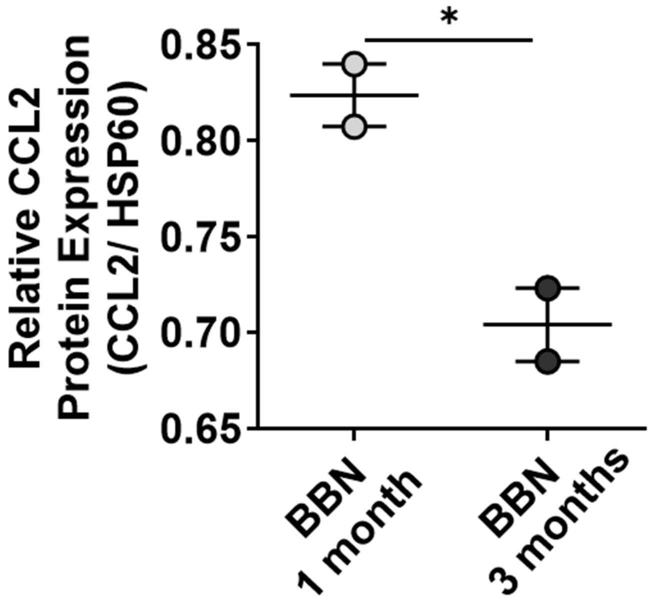

(**C**)

Supplementary Figure S9. CCL2 is downregulated in mouse and human BCa. (**A**) Gene expression profiling interactive analysis (GEPIA) with TCGA BLCA and GTEx datasets was used to calculate differences in CCL2 expression in bladder tumor samples (n = 404) compared with normal bladder samples (n = 28). The red box represents the cancer tissue group, the gray box represents the normal tissue group, and the asterisk represents $p < 0.01$. The dots represented expression in each sample. (**B**) WT mice were challenged orthotopically with 8×10^4 MB49 cells and sacrificed after ~3 weeks for bladder tumor processing. In total, 0.5×10^6 bladder cells were plated in 50 μL in a 96-well plate. The super-

natant was collected after 24 h and CCL2 level was measured by a mouse chemokine array. Relative CCL2 protein expression was normalized with HSP60 control protein expression. (**C**) WT C57BL/6 mice were given BBN in drinking water for 1 month (control) and 3 months (tumor induction by chronic exposure). Mice were sacrificed, bladders were processed and 0.5×10^6 bladder cells were plated in 50 μL in a 96-well plate. The supernatant was collected after 24 h and the CCL2 level was measured by a mouse chemokine array. Relative CCL2 protein expression was normalized with HSP60 control protein expression. Mean ± SEM; * $p < 0.05$, ** $p < 0.01$, unpaired *t*-test.
